# Captive individuals of endangered Philippine raptors maintain native feather mites (Acariformes: Pterolichoidea) species

**DOI:** 10.1016/j.ijppaw.2018.03.002

**Published:** 2018-03-17

**Authors:** Sergey V. Mironov, Boris D. Efeykin, Jayson C. Ibanez, Anna Mae Sumaya, Oleg O. Tolstenkov

**Affiliations:** a Zoological Institute, Russian Academy of Sciences, Universitetskaya Embankment 1, Saint Petersburg, 199034, Russia; b A.N. Severtsov Institute of Ecology and Evolution, Russian Academy of Sciences, Leninskij Prosp. 33, Moscow, 119071, Russia; c Kharkevich Institute for Information Transmission Problems, Russian Academy of Sciences, Bol'shoi Karetnyi per. 19, Moscow, 127051, Russia; d University of the Philippines in Mindanao, Barangay Mintal, Davao City 8022, Philippines; e Philippine Eagle Foundation, Malagos, Baguio District, Davao City 8000, Philippines; f Johann Wolfgang Goethe-University Frankfurt, Buchmann Institute for Molecular Life Sciences and Institute of Biophysical Chemistry, Max-von-Laue-Str. 15, D-60438 Frankfurt, Germany

**Keywords:** Parasites of endangered species, Ectoparasites, Birds of prey, Feather mites, Pterolichoidea, *Pithecophaga jefferyi*, Great philippine eagle, *Nisaetus pinskeri*, Mindanao hawk-eagle, Molecular phylogeny

## Abstract

Endangered species of hosts are coupled with endangered species of parasites, which share the risk of co-extinction. Conservation efforts sometimes include breeding of rare species in captivity. Data on parasites of captive populations of endangered species is scarce and the ability of small numbers of captive host individuals to support the biodiversity of native parasites is limited. Examination of ectosymbionts of the critically endangered Philippine eagles and the endangered Mindanao Hawk-Eagle kept at the Philippine Eagle Center, Philippines, revealed three feather mite species despite regular treatment with insecticide powder. No other ectosymbiont taxa were detected. Studies in morphology and molecular phylogeny of these feather mites based on mitochondrial and nuclear DNA markers indicate that species found were typical for Accipitridae. Three new pterolichoid feather mite species (Acari: Pterolichoidea) were described from two species of eagles (Accipitriformes: Accipitridae) endemic to the Philippines: *Hieracolichus philippinensis* sp. n. (Gabuciniidae) and *Pseudalloptinus pithecophagae* sp. n. (Pterolichidae) from the Great Philippine Eagle *Pithecophaga jefferyi* Ogilvie-Grant, 1896, and *Pseudogabucinia nisaeti* sp. n. (Kramerellidae) from the Mindanao Hawk-Eagle *Nisaetus pinskeri* Gould, 1863. The presence of *H. philippinensis* on *P. jefferyi* supports the recent finding that the Great Philippine Eagle belongs to the lineage of serpent eagles (Circaetinae) rather than to the Harpy and other eagles.

## Highlights

• Small captive groups of endangered birds can maintain native feather mite populations for decades despite regular antiparasitic treatment.
• Our study is a first record of feather mites from endemic raptors or diurnal birds-of-prey in the Philippines, with three new species described.
• Feather mites of the Great Philippine Eagle support the attribution of the host to the Circaetinae rather than to Harpiinae birds of prey lineages.

## Graphical abstract

**Figure:**
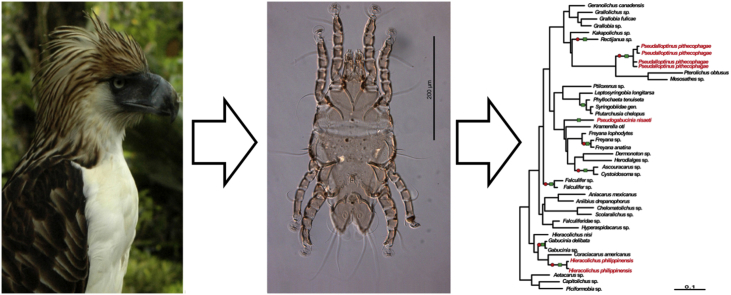

## 1 Introduction

Parasites represent an important component of the ecosystem (Hudson et al., 2006) and support the diversity of the host populations by exerting selective pressure upon their hosts (Dawkins, 1990; Rózsa, 1992). Parasites of endangered species encounter a dual problem. On the one hand, parasites may negatively affect the natural and captive populations of their hosts threatened with extinction (De Castro and Bolker, 2005; McCallum and Dobson, 1995), and on another hand, these parasites often represent endangered species by themselves (Gomez and Nichols, 2013; Rózsa and Vas, 2014). The latter is especially relevant for host-specific parasites (symbionts), such as many ectosymbionts of birds and mammals that often face co-extinction with their host (Buckley et al., 2012).

Host populations of small size harbor reduced diversity of symbiont species due to the parasite loss (Altizer et al., 2007; Lloyd-Smith et al., 2005). The case of the Great Philippine Eagle *Pithecophaga jefferyi* Ogilvie-Grant, 1896 represents an extreme of minimal population size, both because of being a naturally uncommon apex predator in the islands and of current environmental change and habitat fragmentation, with an estimated 250-750 individuals in total (IUCN, 2017). The extremely low number of Philippine eagles increases the probability of loss for their parasites. Moreover keeping and breeding of rare bird species in captivity for the conservation purposes is also accompanied by the loss of their ectosymbionts mostly due to the antiparasitic treatment (Dunn et al., 2009). Therefore, the survival of the ectosymbionts on the captive group of Philippine eagles was under the question. Besides, its position as apex predator in the ecosystem could facilitate Philippine eagles to adopt alien parasite species from its prey. We tested whether the captive individuals of the Philippine raptors maintained the ectosymbionts and if the ectosymbionts found represented the native fauna of the Philippines eagles studied.

No data on parasites for critically endangered Philippine eagles was available so far; therefore, the study of biodiversity of ectosymbionts in these birds represents an essential need. During ectosymbionts examination of captive Philippine eagles in the Philippine Eagle Center, feather mites were found in spite of the annual antiparasitic treatment (dusting the body, wings and the tail with the powder containing carbamates, Gamma powder, a local producer).

Diurnal birds of prey (Accipitriformes and Falconiformes), a group containing the Philippine eagles, are of the most poorly explored major groups of recent birds in relation to their specific feather mite fauna (Astigmata: Analgoidea and Pterolichoidea). Most collections of feather mites from raptors, especially rare species, have been made from museum skins (Gaud, 1983a; b; Gaud and Atyeo, 1996). Nowadays most species of raptors are endangered and highly protected; therefore, they are not easily accessible for parasitological examinations. All data on parasite-host associations of feather mites and raptors published before the end of 20th century were summarized by Philips (2000). After that, just a few papers on mites from raptors have been published (Dabert and Mironov, 2015; Hernandes, 2017; Mironov et al., 2007; Mironov and Galloway, 2003, 2014; Pedroso et al., 2015; Proctor et al., 2006).

In the present work, we studied the fauna of feather mites found on two eagles endemic to Philippines based on both morphology and molecular phylogenetic analysis (genes COI, EF-1α, 18S, 28S). We provided descriptions of three species of pterolichoid feather mites and investigated whether these feather mite species likely represent native fauna of Philippine eagles as opposed to species recently acquired through prey-to-host transmission.

## 2 Material and methods

The mite material used in the present study was collected in the Philippine Eagle Center (Davao City, Malagos, The Philippines, 7°11′6.29″N, 125°24′55.17″E) from two species of endemic raptors, the Great Philippine Eagle *Pithecophaga jefferyi* Ogilvie-Grant, 1896 and the Philippine Hawk-Eagle *Nisaetus pinskeri* Gould, 1863, during annual medical examination of birds by OOT in 2016. Parts of the feathers bearing mites were removed using forceps and a magnifying glass, placed in the tube with 96% ethanol and kept at 4 °C for subsequent studies.

### 2.1 Taxonomic study

Some of the collected mites were mounted on microslides in Hoyer's medium according to the standard techniques used for many groups of small acariform mites (Krantz et al.,. 2009). Investigation of mite specimens and drawings were made by SM using a Leica DM 2500 light microscope with differential interference contrast (DIC) and equipped with a camera lucida. Descriptions of new species and measurement methods follow the formats elaborated for corresponding taxonomic groups of mites (Hernandes, 2017; Hernandes and Mironov 2015; Mironov et al., 2007, 2015; Pedroso et al., 2015). General morphological terms and leg chaetotaxy follow Gaud and Atyeo (1996); idiosomal chaetotaxy also follows these authors with corrections for coxal setation by Norton (1998). Descriptions provide the measurements for a male holotype with a range for paratype males in parentheses, and a range for female paratypes. All measurements are in micrometres (μm). Collection data indicate the places of origin and dates of taking of bird individual from nature.

### 2.2 Molecular study

DNA was isolated from specimens fixed in 96% ethanol using Holterman's method (Holterman et al., 2006) with addition of proteinase K and mercaptoethanol in the lysing solution. Sequences of cytochrome oxidase subunit I (COI), elongation factor 1 alpha gene (EF1), partial sequences of 18S and 28S ribosomal DNA subunits 18S and 28S molecular markers were amplified using an EncycloPlus PCR Kit (Evrogen, Russia) with the parameters recommended by the producer on a Biorad T100 amplifier (United States). The sequences of primers used are given in Table 1. Polymerase chain reaction (PCR) products were visualized in gel, cut out, and cleaned using the SV Gel and PCR Clean-Up System kit (Evrogen, Russia). They were then precipitated by ethanol in the presence of ammonium acetate to increase the efficiency of DNA precipitation. DNA sequencing was performed at the Genome Center for Collective Using (Genome, Russia). Molecular markers used and GenBank accession numbers for the sequences of the species studied are presented in Table 2. The sequences were combined and aligned using the ClustalX program after the addition of sequences from the GenBank (Thompson et al., 1997). Subsequently, the sequences were edited using the Genedoc 2.7 program (Nicholas et al., 1997). The phylogenetic trees were reconstructed in the Mr. Bayes 3.2.3 program (Huelsenbeck and Ronquist, 2001) and RaxML (Stamatakis, 2014) in the CIPRES server (Miller et. al., 2010) with the evolutionary model which was selected based on the results of the analysis in jModelTest2 program (Darriba et al., 2015). Sequences of the *Amerodectes turdinus* (GenBank accession number KU203310) and *Amerodectes sp.* (GenBank accession numbers KU202819 and KU202968) were used as outgroups for phylogenetic reconstructions. The genus Amerodectes (Analgoidea: Proctophyllodidae) was selected as an outgroup for the Pterolichoidea feather mites studied because this genus is well defined morphologically and represents another superfamily, Analgoidea, a sister lineage to all pterolichoidean mites used in our analysis. Taxa of feather mites used for phylogenetic analysis, their systematics and hosts are summarized in Table 3.

**Table 1:** Primers used in the study.

Locus	Primers	Authors
Ef1	40.6F ATYGARAARTTYGARAARGARGC	(Cho et al., 1995; Regier, 2008), (Klimov and OConnor, 2008)
	126F GGGMAARGGYTCNTTCAAGT	
	45.71F GTNGSNGTIAAYAARATGGA	
	914R TCGTGRTGCATYTCNACNG	
	1223R_Psor2 AADGTTTCGACGCACATTGG	
	41.21R TGYCTCATRTCDCGVACRGCRAA	
COI	bcdF05 TTTTCTACHAAYCATAAAGATATTGC	(Dabert et al., 2008)
	bcdR04 TATAAACYTCDGGATGNCCAAAAAA	
18S	ACB_18SF AGGGAGAGGCGCATTTATTAG	Authors
	ACB_18SR GCTGGTTGGCATCGTTTATG	
28S	28SV GTAGCCAAATGCCTCGTCA	(Cryan et al., 2000)
	28SX CACAATGATAGGAAGAGCC

**Table 2:** Molecular markers used and GenBank accession numbers for the sequences of the species studied.

Species	Voucher numbers	EF1 sequences numbers	COI sequences numbers	18S sequences numbers	28S sequences numbers
*Pseudalloptinus pithecophagae*	ZISP 7411	MF967007	MG003448	MG001907	MG001914
*Hieracolichus philippinensis*	ZISP 7454	MF967008	MG003449	MG001908	MG001915
*Pseudalloptinus pithecophagae*	ZISP 7391	MF967009	MG003450	MG001909	MG001916
*Hieracolichus philippinensis*	ZISP 7434	MF967010	MG003451	MG001910	MG001917
*Pseudalloptinus pithecophagae*	ZISP 7401	MF967011	MG003452	MG001911	MG001918
*Pseudogabucinia nisaeti*	ZISP 7312	MF967012	MG003453	MG001912	MG001919
*Pseudalloptinus pithecophagae*	ZISP 7371	MF967013	MG003454	MG001913	MG001920

**Table 3:** Species of feather mites used for molecular phylogenetic analysis.

Feather mite species	GenBank accession number	Superfamily	Family	Host species
Amerodectes sp.	KU202819, KU202968	Analgoidea Trouessart and Megnin, 1884	Proctophyllodidae Megnin and Trouessart, 1884	*Vireo hypochryseus* Sclater, 1863
*Amerodectes turdinus* (Berla, 1959)	KU203310	Analgoidea Trouessart and Megnin, 1884	Proctophyllodidae Megnin and Trouessart, 1884	*Catharus fuscescens* Stephens, 1817
Ascouracarus sp.	JQ000778, JQ000475, JQ000167	Pterolichoidea Trouessart and Mégnin, 1884	Ascouracaridae Gaud and Atyeo, 1976	*Strix virgata* Cassin, 1850
Cystoidosoma sp.	JQ000777, JQ000474, JQ000166	Pterolichoidea Trouessart and Mégnin, 1884	Ascouracaridae Gaud and Atyeo, 1976	*Melanerpes aurifrons* Wagler, 1829
Mesosathes sp.	JQ000753, JQ000448	Pterolichoidea Trouessart and Megnin, 1884	Crypturoptidae Gaud, Atyeo and Berla, 1972	*Crypturellus boucardi* Sclater, 1860
Falculifer sp.	JQ000748, JQ000135, JQ000135	Pterolichoidea Trouessart and Mégnin, 1884	Falculiferidae Oudemans, 1908	*Columba flavirostris* Wagler, 1831
Falculifer sp.	JQ000749	Pterolichoidea Trouessart and Mégnin, 1884	Falculiferidae Oudemans, 1908	*Columba oenas* Linnaeus, 1758
Falculiferidae sp.	JQ000751, JQ000138	Pterolichoidea Trouessart and Mégnin, 1884	Falculiferidae Oudemans, 1908	*Scardafella inca* Lesson, 1847
Hyperaspidacarus sp.	JQ000750, JQ000137, JQ000445	Pterolichoidea Trouessart and Mégnin, 1884	Falculiferidae Oudemans, 1908	*Scardafella inca*
*Freyana anatina* (Koch, 1844)	JQ000743, JQ000438	Pterolichoidea Trouessart and Mégnin, 1884	Freyanidae Dubinin, 1951	*Anas platyrhynchos* Linnaeus, 1758
*Freyana lophodytes* Dubinin, 1950	JQ000746, JQ000441, JQ000133	Pterolichoidea Trouessart and Mégnin, 1884	Freyanidae Dubinin, 1951	*Lophodytes cucullatus* Linnaeus, 1758
Freyana sp.	JQ000744, JQ000439	Pterolichoidea Trouessart and Mégnin, 1884	Freyanidae Dubinin, 1951	*Aix sponsa* Linnaeus, 1758
Freyana sp.	JQ000442	Pterolichoidea Trouessart and Mégnin, 1884 Freyanidae Dubinin, 1951	Freyanidae Dubinin, 1951	*Tadorna ferruginea* Pallas, 1764
Aetacarus sp.	JQ000769, EU152516, JQ000465	Pterolichoidea Trouessart and Mégnin, 1884	Gabuciniidae Gaud and Atyeo, 1975	*Geranospiza caerulescens* Vieillot, 1817
Capitolichus sp.	JQ000774, JQ000470	Pterolichoidea Trouessart and Mégnin, 1884	Gabuciniidae Gaud and Atyeo, 1975	*Dryocopus lineatus* Linnaeus, 1766
Capitolichus sp.	JQ000161	Pterolichoidea Trouessart and Mégnin, 1884	Gabuciniidae Gaud and Atyeo, 1975	*Melanerpes aurifrons* Wagler, 1829
*Coraciacarus americanu*s Alzuet, Cicchino and Abrahamovich, 1988	EU152770, JQ000165, JQ000473	Pterolichoidea Trouessart and Mégnin, 1884	Gabuciniidae Gaud and Atyeo, 1975	*Coccyzus americanus* Linnaeus, 1758
*Gabucinia delibata* (Robin, 1877)	JQ000770, JQ000158, JQ000466	Pterolichoidea Trouessart and Mégnin, 1884	Gabuciniidae Gaud and Atyeo, 1975	*Corvus brachyrhynchos* Brehm, 1822
Gabucinia sp.	JQ000771	Pterolichoidea Trouessart and Mégnin, 1884	Gabuciniidae Gaud and Atyeo, 1975	*Cyanocorax sanblasianus* Lafresnaye, 1842
*Hieracolichus nisi* (Canestrini, 1878)	JQ000776, JQ000164, JQ000472	Pterolichoidea Trouessart and Mégnin, 1884	Gabuciniidae Gaud and Atyeo, 1975	*Accipiter nisus* Linnaeus, 1758
*Hieracolichus philippinensis*	MF967008, MF967010, MG001908, MG001910, MG001915, MG001917	Pterolichoidea Trouessart and Mégnin, 1884	Gabuciniidae Gaud and Atyeo, 1975	*Pithecophaga jefferyi*
Piciformobia sp.	JQ000775, JQ000163, JQ000471	Pterolichoidea Trouessart and Mégnin, 1884	Gabuciniidae Gaud and Atyeo, 1975	*Crotophaga sulcirostris* Swainson, 1827
Dermonoton sp.	JQ000742, JQ000437, JQ000129	Pterolichoidea Trouessart and Mégnin, 1884	Kramerellidae Gaud and Mouchet, 1961	*Glaucidium brasilianum* Gmelin, 1788
*Kramerella oti* (Lönnfors, 1937)	JQ000740, JQ000435	Pterolichoidea Trouessart and Mégnin, 1884	Kramerellidae Gaud and Mouchet, 1961	*Asio otus* Linnaeus, 1758
Kramerella sp.	JQ000128, JQ000436	Pterolichoidea Trouessart and Mégnin, 1884	Kramerellidae Gaud and Mouchet, 1961	*Bubo virginianus* Gmelin, 1788
*Pseudogabucinia nisaeti*	MF967012, MG001912, MG001919	Pterolichoidea Trouessart and Mégnin, 1884	Kramerellidae Gaud and Mouchet, 1961	*Nisaetus pinskeri*
*Geranolichus canadensis* Atyeo and Windingstad, 1979	JQ000755, JQ000142, JQ0004501	Pterolichoidea Trouessart and Megnin, 1884	Pterolichidae Trouessart and Megnin, 1884	*Grus canadensis* Linnaeus, 1758
*Grallobia fulicae* (Trouessart, 1885)	JQ000757	Pterolichoidea Trouessart and Megnin, 1884	Pterolichidae Trouessart and Megnin, 1884	*Fulica atra* Linnaeus, 1758
Grallobia sp.	JQ000756	Pterolichoidea Trouessart and Megnin, 1884	Pterolichidae Trouessart and Megnin, 1884	*Porzana carolina* Linnaeus, 1758
Grallolichus sp.	JQ000758, JQ000145, JQ000453	Pterolichoidea Trouessart and Megnin, 1884	Pterolichidae Trouessart and Megnin, 1884	*Gallinula chloropus* Linnaeus, 1758
Kakapolichus sp.	JQ000759, JQ000454	Pterolichoidea Trouessart and Megnin, 1884	Pterolichidae Trouessart and Megnin, 1884	*Nestor notabilis* Gould, 1856
*Pseudalloptinus pithecophagae*	MF967007, MF967009, MF9670011, MF9670013, MG001920, MG001914, MG001909, MG001918	Pterolichoidea Trouessart and Megnin, 1884	Pterolichidae Trouessart and Megnin, 1884	*Pithecophaga jefferyi*
*Pterolichus obtusus* Robin, 1877	JQ000754, EU152513, JQ000449	Pterolichoidea Trouessart and Megnin, 1884	Pterolichidae Trouessart and Megnin, 1884	*Gallus gallus* Linnaeus, 1758
*Aniacarus mexicanus* Gaud and Atyeo, 1990	JQ000762, JQ000457	Pterolichoidea Trouessart and Mégnin, 1884	Pterolichidae Trouessart and Mégnin, 1884	*Crotophaga sulcirostris* Swainson, 1827
*Aniibius drepanophorus* Gaud and Atyeo, 1990	JQ000763	Pterolichoidea Trouessart and Mégnin, 1884	Pterolichidae Trouessart and Mégnin, 1884	*Crotophaga sulcirostris*
Chelomatolichus sp.	JQ000761, JQ000456, JQ000148	Pterolichoidea Trouessart and Mégnin, 1884	Pterolichidae Trouessart and Mégnin, 1884	*Amazona autumnalis* Linnaeus, 1758
Herodialges sp.	JQ000752, JQ000447, JQ000139	Pterolichoidea Trouessart and Mégnin, 1884	Pterolichidae Trouessart and Mégnin, 1884	*Ardea Herodias* Linnaeus, 1758
Scolaralichus sp.	JQ000760, JQ000455, JQ000147	Pterolichoidea Trouessart and Mégnin, 1884	Pterolichidae Trouessart and Mégnin, 1884	*Amazona autumnalis*
*Aniibius drepanophorus* Gaud and Atyeo, 1990	JQ000458	Pterolichoidea Trouessart and Mégnin, 1884	Pterolichoidae Trouessart and Mégnin, 1884	*Crotophaga sulcirostris*
*Grallobia fulicae* (Trouessart, 1885)	JQ000144	Pterolichoidea Trouessart and Mégnin, 1884	Pterolichoidae Trouessart and Mégnin, 1884	*Fulica atra*
Ptiloxenus sp.	JQ000764, JQ000460, JQ000152	Pterolichoidea Trouessart and Megnin, 1884	Ptiloxenidae Gaud, 1982	*Podiceps auritus* Linnaeus, 1758
Rectijanua sp.	EU152767, JQ000459	Pterolichoidea Trouessart and Megnin, 1884	Rectijanuidae Gaud, 1961	*Aix sponsa* Linnaeus, 1758
*Leptosyringobia longitarsa* (Megnin and Trouessart, 1884)	JQ000767, JQ000155, JQ000463	Pterolichoidea Trouessart and Megnin, 1884	Syringobiidae Trouessart, 1896	*Pluvialis squatarola* Linnaeus, 1758
*Phyllochaeta tenuiseta* Dabert and Atyeo, 1993	JQ000768, JQ000464, JQ000156	Pterolichoidea Trouessart and Megnin, 1884	Syringobiidae Trouessart, 1896	*Charadrius vociferus* Linnaeus, 1758
Syringobiidae sp.	JQ000766, JQ000154	Pterolichoidea Trouessart and Megnin, 1884	Syringobiidae Trouessart, 1896	*Calidris minuta* Leisler, 1812
*Plutarchusia chelopus* Oudemans, 1904	JQ000765, JQ000461, JQ000153	Pterolichoidea Trouessart and Mégnin, 1884	Syringobiidae Trouessart, 1896	*Tringa totanus* Linnaeus, 1758
Syringobiidae sp.	JQ000464	Pterolichoidea Trouessart and Mégnin, 1884	Syringobiidae Trouessart, 1896	*Charadrius vociferus*

We tested the congruence of operational taxonomic units (OTUs) by the application of two analytical methods: Generalized Mixed Yule Coalescent (GMYC) (Pons et al., 2006) and Automatic Barcode Gap Discovery (ABGD) (Puillandre et al., 2012). GMYC represents a model-based approach, aiming to discover the maximum likelihood solution for the threshold between the branching rates of speciation, while ABGD detects the statistically inferred barcode gap - difference between the greatest intraspecific distance and the smallest interspecific distance - and uses it to partition the data.

Depositories of type material and voucher specimens used for molecular study are as follows: UMICHZ — Museum of Zoology of the University of Michigan, Ann Arbor, USA; ZISP — Zoological Institute of the Russian Academy of Sciences, Saint Petersburg, Russia.

## 3 Results

### 3.1 Systematics

Superfamily Pterolichoidea Trouessart et Mégnin, 1984

Family Gabuciniidae Gaud and Atyeo, 1975

Genus *Hieracolichus* Gaud and Atyeo, 1975

Type species: *Pterolichus nisi* Canestrini, 1878, by original designation.

Representatives of the genus *Hieracolichus*, currently including nine species, are restricted to birds of the order Accipitriformes (Gaud, 1983b; Gaud and Atyeo, 1974; Hernandes, 2017). Of them, seven *Hieracolichus* species are known from African raptors (Gaud, 1983a). Although the genus *Hieracolichus* is not species-rich, taxonomic limits and species content of this genus need a revision (Mironov et al., 2007). This genus is very close to the genus *Aetacarus* Gaud and Atyeo, 1975, which has 10 of 12 known species associated with Accipitriformes. The genera *Aetacarus* and *Hieracolichus* differ from each other based only on a single feature of females: in *Hieracolichus,* coxal setae *4a* are situated slightly anterior to the genital papillae and close to genital setae *g*, while in *Aetacarus*, these setae are situated posterior to the genital papillae, in some species even posterior to coxae IV. Because of a weak morphological boundary between two genera, Gaud (1983b) was unable to create separate keys to them and provided a single key where species of these genera were mixed together. Position of some species currently referred to the genus Hieracolichus is questionable. Thus, *Hieracolichus hirundo* (Mégnin and Trouessart, 1884) placed in this genus by Gaud and Atyeo (1975) and recently redescribed by Hernandes (2017) should be formally referred to the genus Aetacarus. The redescription of this species clearly shows that in females, setae g are closer to setae 4b than 4a, and the genital papillae are situated anterior to setae 4a. These are the two main diagnostic features of Aetacarus distinguishing it from Hieracolichus. Referring of *H. ostudus* Gaud, 1978 to Hieracolichus, being the only species of this genus having inflated bases of epimerites I and II and lacking solenidion σ on genu III, is also doubtful.

Type material. Male holotype (ZISP 7412), 13 male and 9 female paratypes from *Pithecophaga jefferyi* Ogilvie-Grant, 1896 (Accipitridae), THE PHILIPPINES, Agusan del Norte, Santiago, Mt. Mamajao near Lake Mainit, caught on April 1974, mite collector O.O. Tolstenkov. The bird was at least 42 years old in 2016 when the mites were sampled. Voucher specimen: paratype female ZISP 7434.

Depository: holotype, 8 male and 5 female paratypes, including voucher – ZISP, remaining paratypes – UMICHZ.

Additional material. 3 males, 1 females from *P. jefferyi*, THE PHILIPPINES, Lanao del Sur, Wao, wild-caught on 25 April 2015, mite collector O.O. Tolstenkov. Voucher specimen: female ZISP 7454.

### 3.2 Description

**Fig. 1:**
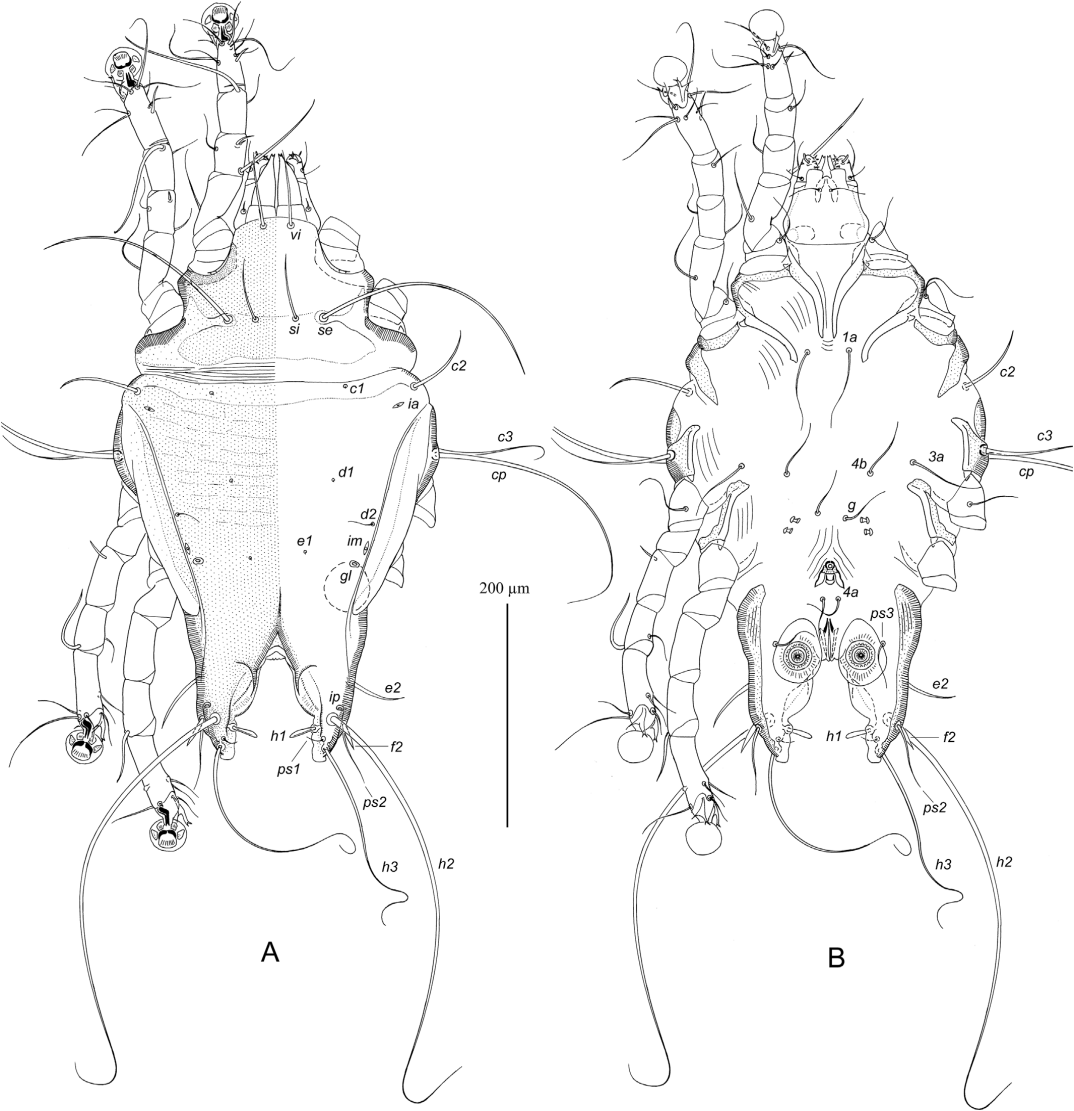
*Hieracolichus philippinensis* sp. n. male. A – dorsal view, B – ventral view.

MALE (Fig. 1, Fig. 3D). (Holotype, range for nine paratypes in parentheses). Gnathosoma roughly trapezoidal, length including palps 80 (75–83), greatest width at base 78 (75–78). Idiosoma length from anterior end to bases of setae *h3* on lobar apices 475 (465–490), greatest width at level of humeral setae 290 (270–290); length of hysterosoma 340 (330–350). Prodorsal shield: occupying almost entire prodorsum, Prodorsal shield: antero-lateral extensions protruding to margins of propodosoma between trochanters I and II and fused with epimerites Ia, antero-lateral margins heavily sclerotized, lateral margins with narrow and deep incisions encircling bases of setae *se*, posterior margin slightly sinuous, greatest length 135 (120–135), width at posterior margin 180 (170–180). Setae *vi* spiculiform, 70 (67–73) long, extending slightly beyond tips of palps. Setae *si* spiculiform, 57 (55–60) long. Distance between bases of scapular setae: *se:se* 87 (78–85), *si:si* 37 (28–35). Subhumeral setae *c3* filiform, with lanceolate enlargement in basal 1/3, 100 (95–105) long. Hysteronotal shield: greatest length from anterior margins to bases of setae *h3* 330 (320–345), length along midline 230 (225–240), width at anterior margin 155 (150–160), anterior margin slightly concave, surface of anterior half with sparse transverse striation. Lateral bands distinct. Lobar areas of hysteronotal shield not separated from main body of hysteronotal shield. Supranal concavity small triangular. Setae *c2* thin spiculiform, 70 (70–70) long, situated in anterior angles of hysteronotal shield, cupules *ia* immediately postero-mesal to their bases. Setae *e1* situated at level of hysteronotal gland openings *gl* or slightly anterior to them. Length of terminal cleft from anterior end to lobar apices (setae *h3*) 93 (90–100), greatest width at level of setae *h1* 67 (65–75). Margin of anterior one third of terminal cleft heavily sclerotized, margin of remaining part membranous; this membranous margin strongly convex anterior to bases of setae *h1*, posterior ends of opisthosomal lobes with small semi-ovate extensions. Setae *e2* spiculiform 52 (50–58) long, with apices extending slightly beyond level of setae *h2*; setae *f2* narrowly lanceolate, 27 (27–32) long, situated at level of setae *h2*, setae h1 lanceolate with rounded apex, 23 (22–25) long, 3.5 (3.5–5) wide, situated posterior to level of setae *h2*. Distances between bases of dorsal setae and gland openings: *c2:d2* 120 (100–115), *d2:e2* 140 (140–150), *e2:h3* 60 (60–68), *d2:gl* 32 (29–35), *h3:h3* 95 (95–105), *h2:h2* 108 (100–115), *d1:d2* 37 (25–37), *e1:e2* 110 (105–115).

Epimerites I, II without inflated bases. Epimerites I with tips simple, not extending to bases of coxal setae *1a*. Epimerites II slightly curved. Genital apparatus at level of trochanters IV, 23 (22–25) × 25 (25–30), aedeagus not extending to its base. Bases of setae *4a* separated. Setae *4b* are slightly posterior to level of setae *3a*. Setae *g* at level of anterior pair of genital papillae. Distances between ventral setae: *4b:g* 37 (35–42), *g:4a* 75 (67–75), *4a:ps3* 37 (37–42), *ps3:h3* 93 (87–98), *4a: 4a* 15 (13–16). Anal suckers 25 (22–25) in diameter, corolla with 18–19 rounded denticles.

Femora I, II without ventral crest. Seta *cG* of genu I spiculiform, 90 (85–90) long, slightly exceeding entire length of genu and tibia. Solenidion *σ1* of genu I 8 (8–11) long, much longer than solenidion *σ2*. Solenidion σ of genu III situated in basal part of this segment. Solenidion *φ* of tibia IV s shorter than corresponding tarsus. Tarsus IV with seta *d* button like and seta *e* of minute spine-like. Legs IV with distal half of tarsus extending beyond level of lobar apices. Length of tarsi: I, II 22 (22–24), III, IV 24 (22–25). Ambulacral disc of tarsus I ovate and in longitudinal diameter noticeably longer than the more circular-shaped ambulacral discs of tarsi II–IV. Length of tarsi: I 45 (45–50), II 58 (56–59), III 62 (60–63), IV 68 (65–68). Length of solenidia: *σ1*I 18 (13–18), *σ*II 6 (5–7), *σ*III 20 (15–20), *ω1*I 12 (11–14), *ω1*II 25 (24–26).

**Fig. 2:**
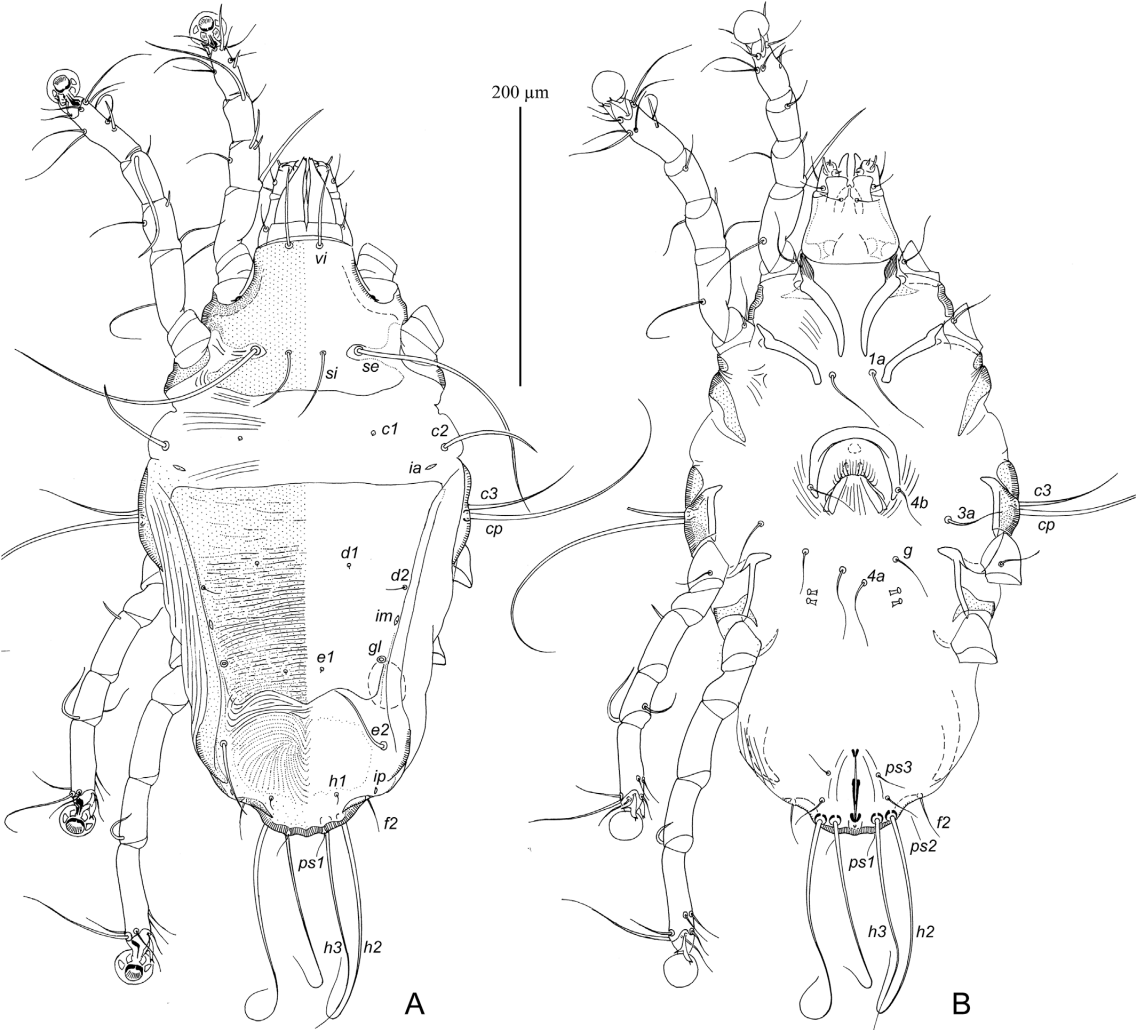
*Hieracolichus philippinensis* sp. n. female. A – dorsal view, B – ventral view.

**Fig. 3:**
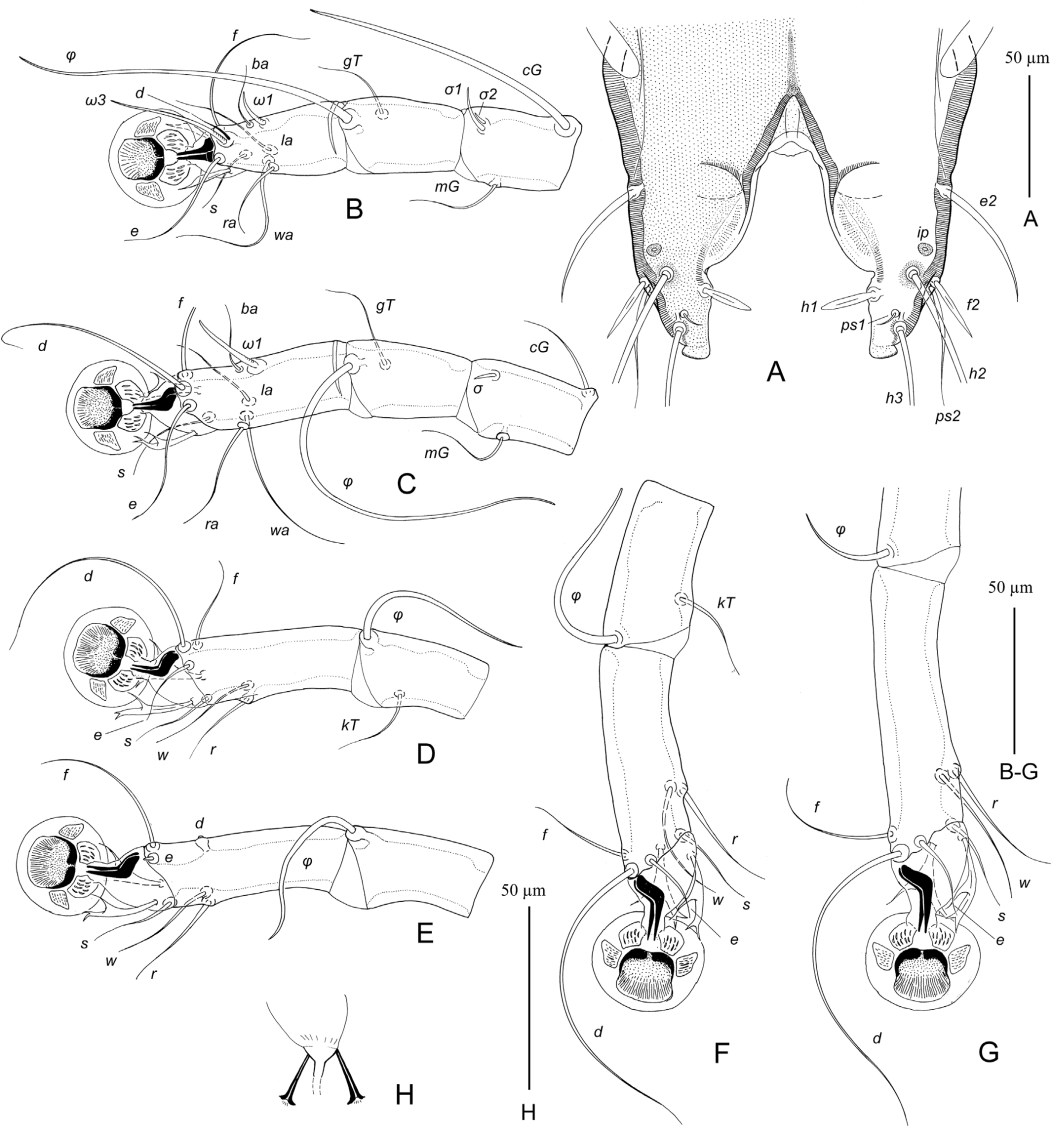
*Hieracolichus philippinensis* sp. n. details. A – opisthosoma of male, dorsal view B–D – genua, tibiae and tarsi I–III of male, respectively, E – tibia and tarsus IV of male, G – tibia and tarsus IV of female, H – spermatheca and spermaducts.

FEMALE (Fig. 2, Fig. 3E, F) (range for nine paratypes). Gnathosoma, length × width, 105–110 × 100–102. Idiosoma, length × width, 615–640 × 340–355, length of hysterosoma 430–445. Prodorsal shield shaped as in male, 155–165 × 185–200. Setae *vi* spiculiform, 75–80 long, barely reaching tips of palps. Setae *si* spiculiform, 75–78 long. Distance between bases of scapular setae: *se:se* 105–120, *si:si* 35–38. Subhumeral setae *c3* filiform, 115–125 long. Hysteronotal shield: main body with almost straight anterior margin, anterior angles acute, posterior end extending to midlevel between hysteronotal gland openings *gl* and setae *e2*, posterior margin with blunt-angular median extension and pair of shallow concavities, greatest length 360–370, width at anterior margin 270–280, surface with faint transverse striation. Setae *c2* spiculiform, 92–105 long, situated off hysteronotal shield; cupules *ia* postero-mesal to them and also off this shield. Setae *d2* short filiform, about 20 long. Setae *e1* approximately at level of hysteronotal gland openings *gl*. Lateral bands well developed, longer than main body of hysteronotal shield, with posterior ends almost extending to cupules *ip* and slightly curved medially. Posterior one quarter of opisthosoma poorly sclerotized, with fine striation and, in some specimens, with barely distinct punctation. Setae *e2* spiculiform, 115–125 long, setae *f2* filiform 30–40 long, setae *h1* short filiform, about 10 long; both pair situated on poorly sclerotized area of opisthosoma. Posterior end of opisthosoma with wide and rounded median extension bearing setae *h2, h3* and *ps1* and with strongly sclerotized margin. Distances between dorsal setae and gland openings: *c2:d2* 135–155, *d2:e2* 155–170, *e2:h3* 78–83, *d2:gl* 72–78, *h1:h1* 62–70, *h2:h2* 75–80. *h3:h3* 45–48.

Epimerites I, II without basal inflation. Epimerites I not extending to setae *1a*. Epigynum horseshoe-shaped, 72–88 long, 92–100 wide. Setae *4b* situated on epigynum, close to its tips. Setae *4a* situated slightly anterior to genital papillae. Copulatory opening immediately posterior to anal opening. Distances between ventral setae: *4b:g* 75–80, *4b:3a* 35–50, *g:4a* 13–25, *ps2:ps3* 27–32, *ps2:ps2* 67–72*.*

Femora I, II with ventral crest. Setae *cG* of long spiculiform, 22–28 long, approximately subequal to entire length of genu and tibia I. Legs IV with tarsus and distal part of tibia extending beyond posterior end of opisthosoma. Length of tarsi: I 53–58, II 72–78, III 75–80, IV 93–100. Length of solenidia: *σ1*I 23–28, *σ*II 8–12, *σ*III 17–25, *ω1*I 16–18, *ω1*II 22–24.

**Differential diagnosis**. Among previously described species, *Hieracolichus philippinensis* sp. n. is more similar to *H. dobyi* Gaud and Mouchet, 1959 described from *Stephanoaetus coronatus* (Linnaeus, 1766) in Africa (Gaud and Mouchet, 1959; Gaud, 1983b) in having, in males, setae *e2* extending to the level of setae *h2* and *f2*, and relatively short and narrowly lanceolate setae *h1*. *Hieracolichus philippinensis* differs from this species by the following features: in both sexes, setae *c3* are long, filiform and exceed 100 μm in length, and genual solenidion σ is situated at the base of genu III; in males, setae *g* are situated almost at the level of anterior genital papillae; setae *h1* are short (22–25 μm), and the inner margins of opisthosomal lobes have a pair of noticeably convex membranes in the anterior part of the terminal cleft; in females, the hysteronotal shield is shaped as an inverted trapezium and the posterior one third of the opisthosoma is devoid of sclerotization except the posterior margin, and tarsus IV completely extends beyond the posterior margin of the opisthosoma. In both sexes of *H. dobyi*, setae *c3* are narrowly lanceolate at base with filiform apex (80-90 μm long), and genual solenidion σ is situated at the midlength of genu III; in males, setae *g* are situated anterior to the level of genital papillae; setae *h1* are narrowly lanceolate, curved and 30–35 μm long, and the inner margins of opisthosomal lobes are almost straight; in females, the hysteronotal shield is shaped as an inverted trapezium and the posterior one third of the opisthosoma is devoid of sclerotization except for the posterior margin, and tarsus IV slightly (by ¼ the length) extends beyond the posterior margin of the opisthosoma.

**Etymology**. The specific epithet is derived from the country, where the mite was found.

Family Pterolichidae Trouessart et Mégnin, 1884

Subfamily Pterolichinae Trouessart et Mégnin, 1884

Genus Pseudalloptinus Dubinin, 1956

Type species: Pterolichus (Pseudalloptes) aquilinus var. milvulinus Trouessart, 1884, by original designation.

The genus *Pseudalloptinus* originally included pterolichine mites associated with birds from the orders Accipitriformes, Falconiformes, Gruiformes, Ciconiiformes and Psittaciformes (Dubinin, 1956; Gaud and Mouchet, 1959). After a revision (Gaud, 1988), the content of this genus was reduced to five species associated exclusively with birds of the order Accipitriformes. The genus *Pseudalloptinus* is readily distinguishable from other pterolichine genera in having, in most species, a unique structure in males: the postgenital sclerite [ = fossette post-genitale of Gaud (1988)]. This sclerite, being apparently a derivative of adanal apodemes, is situated between the genital apparatus and anal field and usually is stirrup-shaped or roughly ovate.

Type material. Male holotype (ZISP 7330), 20 male and 20 female paratypes from Pithecophaga jefferyi Ogilvie-Grant, 1896 (Accipitridae), THE PHILIPPINES, Lanao del Sur, Wao, 25 April 2015, mite collector O.O. Tolstenkov. Voucher specimen: female paratype ZISP 7371.

Depository. Holotype, 15 male and 15 female paratypes – ZISP, remaining paratypes – UMICHZ.

Additional material. 20 males, 20 females from 3 *P. jefferyi* individuals originated from the following locations: 10 males, 10 females – THE PHILIPPINES, Agusan del Norte, Santiago, Mt. Mamajao near Lake Mainit, caught on April 1974; 5 males, 5 females, THE PHILIPPINES, Davao Oriental, Mati, Don Salvador, South Biasong, caught on 13 January 2011; 5 males, 5 females, THE PHILIPPINES, Davao City, Malagos, Philippine Eagle Center, 4 February 2002 (captive breed), mite collector O.O. Tolstenkov. Voucher specimens: female paratypes ZISP 7391, 7411.

**Fig. 4:**
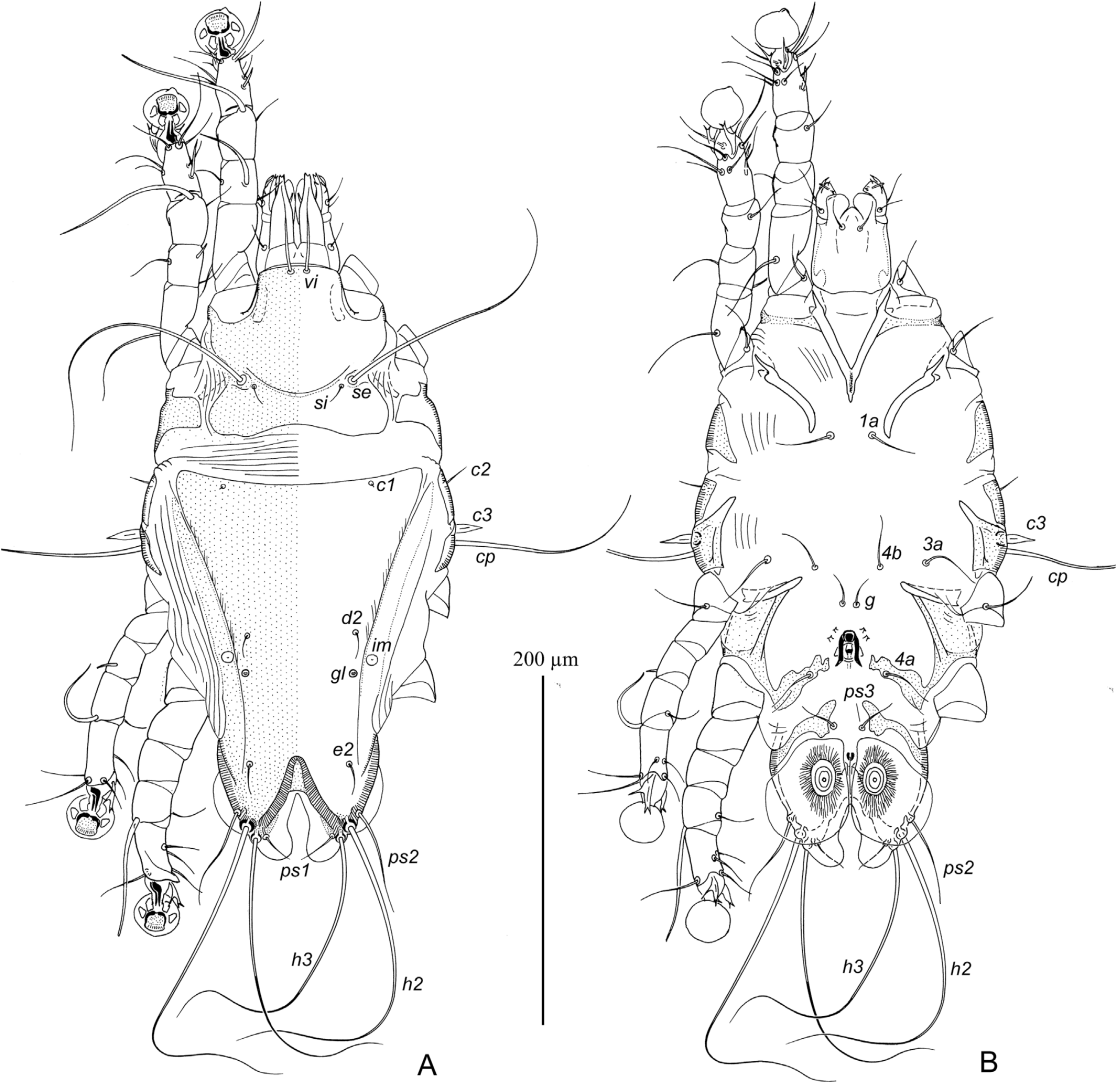
*Pseudalloptinus pithecophagae* sp. n. male. A – dorsal view, B – ventral view.

MALE (Fig. 4, 6A–C). (Holotype, range for eight paratypes in parentheses). Gnathosoma: length including palps 62 (60–65), greatest width at base 47 (46–50). Idiosoma length from anterior end to lobar apices (bases of setae *h3*) 25 (325–350), greatest width at level of humeral setae 180 (180–195). Length of hysterosoma 215 (210–225). Prodorsal shield: occupying most part of prodorsum, antero-lateral extensions acute, lateral margins with deep and narrow extensions encircling bases of scapular setae *se*, posterior margin slightly concave, length along midline 98 (95–105), greatest width 102 (100–110). Setae *vi* filiform, 38 (28–38) long, not extending to palpal apices. Setae *se* separated by 65 (65–68). Setae *si* minute filiform, close to bases of corresponding setae *se*. Scapular and humeral shields present. Setae *c2* filiform, 15 (12–15) long, situated on anterior margin of humeral shields. Subhumeral setae *c3* lnceolate 20 (18–20) long, 4 (3.7–5) wide.

Hysteronotal shield: greatest length from anterior margins to bases of setae *h3* 212 (210–215), width at anterior margin 145 (140–150), anterior margin slightly concave, surface without ornamentation. Lateral bands distinct, narrow. Hysteronotal gland opening *gl* situated at level of trochanters IV. Setae *d2* minute filiform about 10 (10–12) long; setae *e2* filiform, 16 (15–18) long, situated at level of anterior end of supranal concavity, not extending to lobar apices. Opisthosomal lobes roughly triangular, at base slightly wider than long, rounded posteriorly. Terminal cleft roughly semi-ovate, 28 (25–30) long, 35 (34–38) in width at level of setae *ps1*. Supranal concavity open posteriorly into terminal cleft. Terminal cleft with narrow entire membrane forming semi-ovate terminal extensions on lobar apices, length of these extensions 10 (10–15) long, wide at base 18 (17–20). Setae *ps2* long filiform, extending far beyond level of lobar apices; setae *ps1* minute filiform, about 15 long, situated near bases of setae *h2*. Distances between dorsal setae: *c2:d2* 87 (80–88), *d2:e2* 75 (72–80), *e2:h3* 42 (40–45), *ps1:ps1* 40 (38–42), *h2:h2* 60 (60–65), *h3:h3* 50 (50–55), *ps2:ps2* 70 (70–75).

Epimerites I fused into a Y with short stem. Epimerites IIa present. Genital apparatus situated at level of anterior margin of trochanters IV, 14 (14–15) long, 14 (13–17) wide. Setae *4b* slightly posterior to level of setae *3a*. Setae *g* equidistant from genital arch apex and level of setae *4b*. Anterior genital papillae at level of genital arch apex. Epimerites IVa long, bearing bases of setae *4a* near tips and flanking base of genital arch. Adanal apodemes with L-shaped inner ends flanking median area with bases of setae ps3 but not forming separate postegenital sclerite. Anal suckers 13 (13–15) in diameter, corolla without indentation, surrounding membrane very wide and extending laterally over lateral margins of opisthosoma. Distances between ventral setae: *4b:g* 23 (22–25), *g:4a* 40 (40–47); *4a:ps3* 30 (30–32), *ps3:h3* 67 (65–68).

Setae of tarsi I, II filiform. Solenidion *σ1* situated at its midlevel of genu I and 1.3–1.5 times longer than this segment. Genual setae *cG*I, *cG*II, *mG*I and *mG*II filiform, shorter than corresponding segments. Solenidion *σ* of genu III in distal part of segment. Legs IV with distal half of tarsus extending beyond level of lobar apices. Tarsus IV with claw-like apical extension, setae d and e minute are absent. Solenidion *φ* of tibia IV about 1.5 times longer than tarsus IV. Length of tarsi: I 35 (35–37), II 35 (35–38), III 38 (37–40), IV 33 (32–34). Length of solenidia: *σ1*I 40 (40–45), *σ*II 8 (7.5–8), *σ*III 8 (8–10), *ω1*I 11 (10–03), *ω1*II 18 (16–18).

**Fig. 5:**
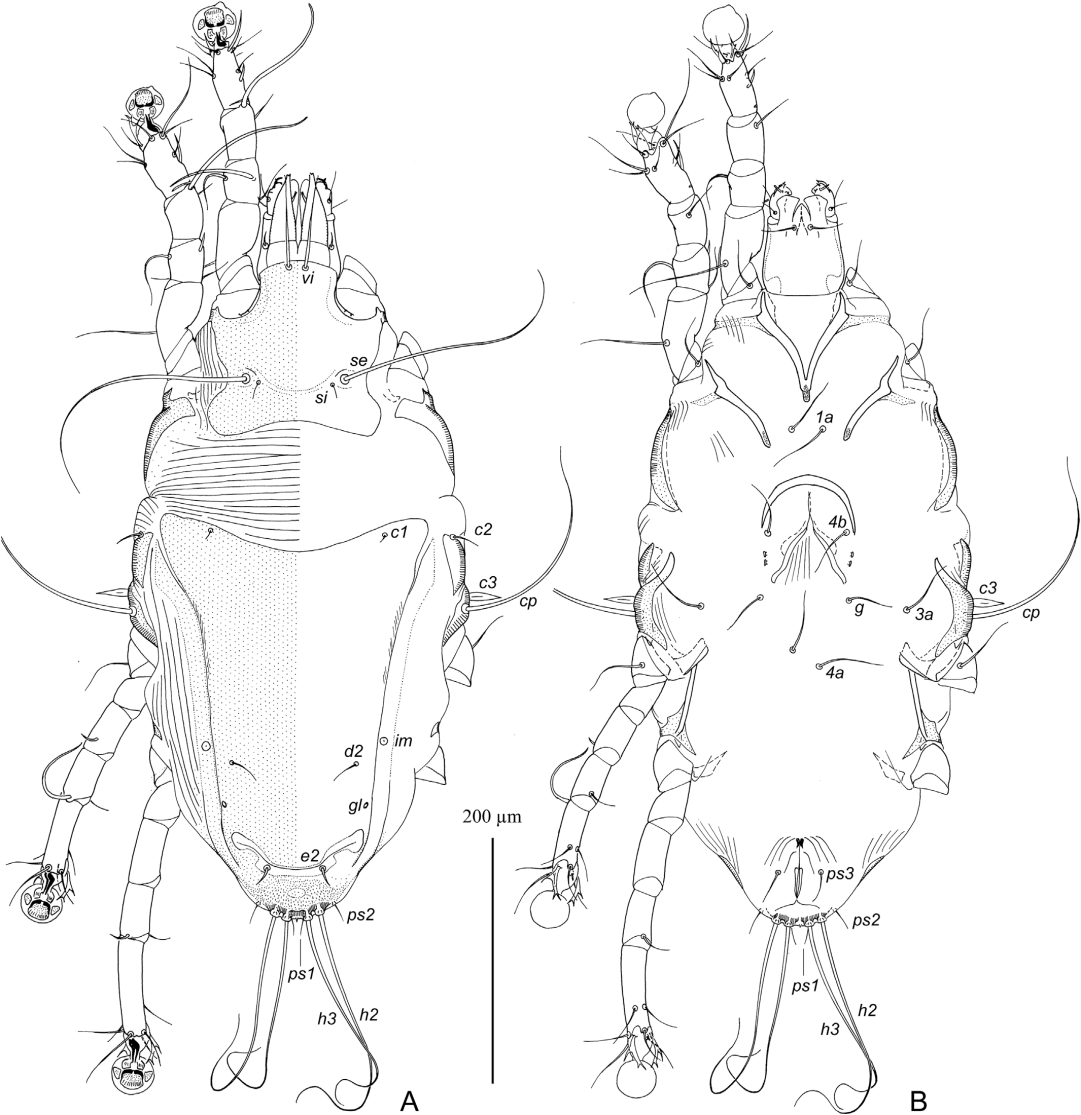
*Pseudalloptinus pithecophagae* sp. n. female. A – dorsal view, B – ventral view.

**Fig. 6:**
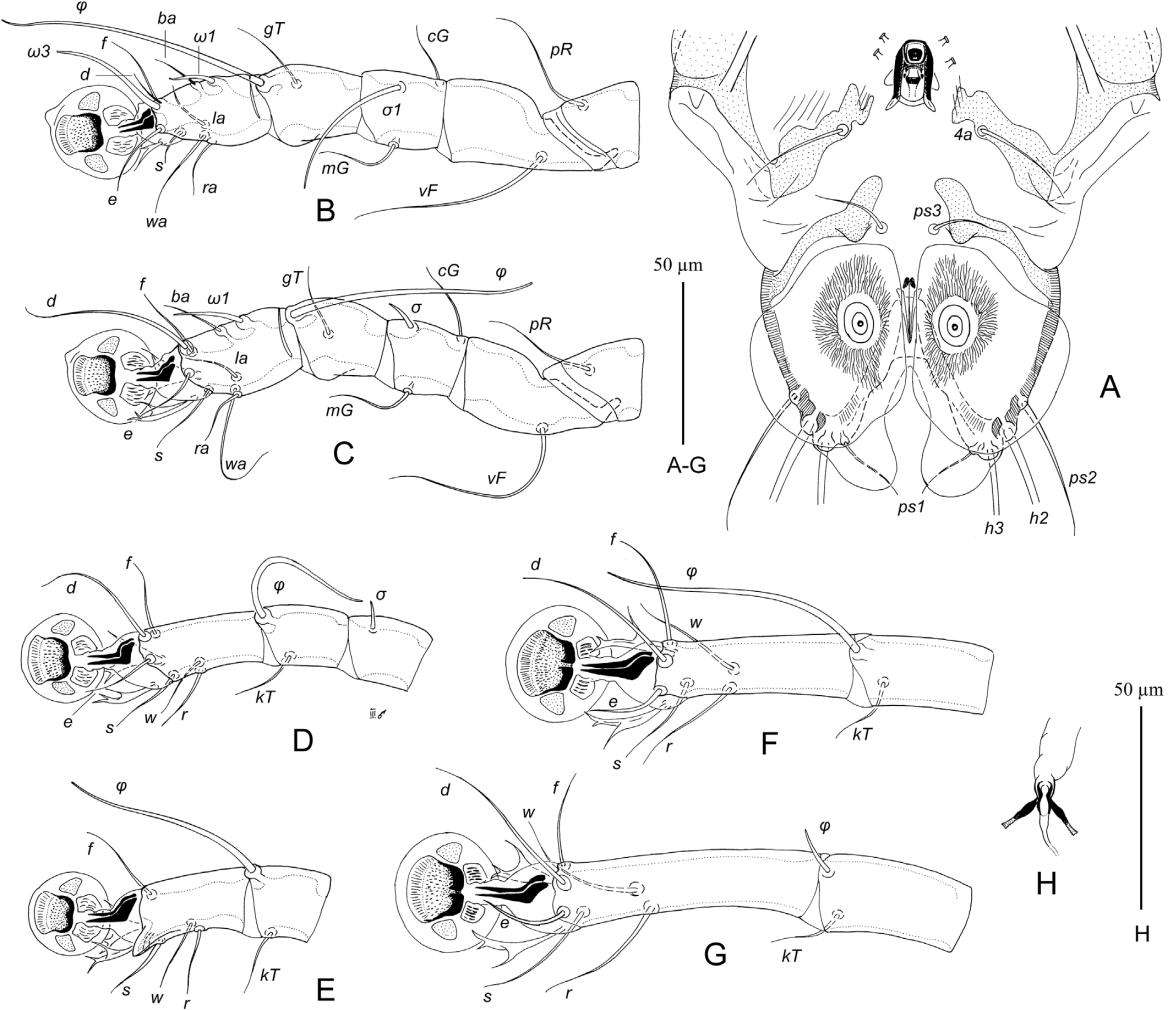
*Pseudalloptinus pithecophagae* sp. n. details. A – opisthosoma of male, ventral view, B–D – legs I–III of male, respectively, E – tibia and tarsus IV of male, G – tibia and tarsus IV of female, H – spermatheca and spermaducts.

FEMALE (Fig. 5, Fig. 6 G,H). Gnathosoma, length × width, 82–85 × 67–72. Idiosoma, length × width, 510 × 550. Length of hysterosoma 325–365. Prodorsal shield: shaped as in male, but lateral margins without deep incisions, 135–145 long, 135–140 wide. Setae *se* separated by 80–85; setae *si* minute filiform, situated closely to corresponding setae *se*. Scapular and humeral shields present. Setae *c2* short filiform, 20 (18–20) long, situated in anterior margin of humeral shields. Subhumeral setae *c3* lanceolate, 26–30 long, about half the length of humeral setae *cp*. Hysteronotal shield: entire, extending to posterior end of opisthosoma, anterior margin concave, 300–340 long, 210–220 wide at anterior margin surface without ornamentation, posterior end with desclerotized transverse area bearing setae *e2*. Setae *c1* on hysteronotal shield near its anterior margin. Setae *d2* situated approximately at midlevel between cupules *ip* and hysteronotal gland openings *gl*. Setae *e2* filiform, about 10–12 long. Lateral bands present, poorly distinct. Posterior margin of opisthosoma with relatively wide terminal extension bearing setae *h2, h3* and *ps1*. External copulatory tube minute, situated terminally about 2–3 long. Spermatheca and spermaducts as in Fig. 6H, length of secondary spermaducts 10–12. Length of opisthosomal setae: *e2* 18–20, *f2* 8–10, *ps1* 5–6, *ps2* 15–18. Distances between dorsal setae and openings: *c2:d2* 175–190, *d2:e2* 80–95, *d2:gl* 34–36, *h2:h3* 40–52, *h2:h2* 35–38, *h3:h3* 17–18.

Epimerites I as in male. Epimerites IVa present. Epigynum semicircular, thin, 42–48 long, 65–80 wide, with tips extending to level of setae *4b*. Apodemes of oviporus narrow, barely sclerotized. Setae *g* situated approximately equidistant from levels of setae *4b* and *g*. Distances between ventral setae: *4b:g* 50–58, *4b:3a* 52–65, *g:4a* 38–52.

Legs I–III as in male. Solenidion σ of genu III in distal part of segment. Solenidion φ of tibia III slightly longer than corresponding tarsus; solenidion φ of tibia IV about 1/5 the corresponding tarsus. Legs IV with tarsus and distal half of tibia extending beyond posterior end of opisthosoma. Legs I, as in male. Length of tarsi: I 50–53, II 50–55, III 57–60, IV 78–80. Length of solenidia: *σ1*I 68–80, *σ*II 10–15, *σ*III 10–18, *ω1*I 12-14, *ω1*II 26–28.

**Differential diagnosis**. The new species, *Pseudalloptinus pithecophagae* sp. n. is most similar to *P. africanus* Gaud, 1988 and *P. milvulinus* (Trouessart,1884) in having the following features: in both sexes, setae *c3* are lanceolate; in males, opisthosomal lobes are well developed, with semi-ovate terminal membranes; and in females, the striated sejugal area is large and constitutes about 1/5th of the total length of the idiosoma. *Pseudalloptinus pithecophagae* sp. n. differs from these species by the following features: in males, the genital apparatus is situated at the level of the anterior margin of trochanters IV, epimerites IVa are long and almost extending to the genital arch, and setae *e2* are filiform, situated at the level of the anterior end of supranal concavity and not do not extend to lobar apices; in females, the hysteronotal shield is entire, the epigynum is semicircular and extends to the level of setae *4b*, setae *c1* is situated on the hysteronotal shield, external copulatory tube is minute (only 2–3 μm long), and setae *g* are situated at the level of setae *3a*. In males of *P. africanus* and *P. milvulinus*, the genital apparatus is situated at the level of the posterior margin of trochanters III, epimerites IVa are poorly developed, and setae *e2* are spiculiform, situated posterior to the supranal concavity and extend beyond the lobar apices; in females, the hysteronotal shield is spit into a large anterior piece and a small pygidial fragment covering the very posterior end of the opisthosoma, the epigynum is bow-shaped and does not extend to the level of setae *4b*, setae *c1* are situated on striated tegument near the anterior margin of the hysteronotal shield, the external copulatory tube is about 15 μm long and curved ventrally, and setae g are situated posterior to the level of setae *3a*.

The unique feature of *P. pithecophagae* males, easily discriminating this species from all previously known *Pseudalloptinus* species, is the absence of the entire postgenital sclerite well separated from the adanal apodemes. In this species, L-shaped tips of adanal apodemes turned anteriorly and flank small median area with setae *ps3*, apparently corresponding to the lateral pieces of the postgenital sclerite of other species of this genus.

**Etymology**. The specific epithet is derived from the generic name of the type host and is a noun in the genitive case.

Family Kramerellidae Gaud et Mouchet, 1961

Genus Pseudogabucinia Černy, 1961

Type species: Pterolichus ciconiae Canestrini et Berlese, 1881, by monotypy.

Up to now, the feather mite genus *Pseudogabucinia* has included only five species with hosts erratically distributed among non-passerine orders: Accipitriformes, Ciconiiformes, Falconiformes, Gruiformes, and Otidiformes (Table 4) (Atyeo and Windingstad, 1979; Canestrini and Berlese, 1881; Dubinin, 1956; Gaud, 1968, 1983a; Gaud and Mouchet, 1961; Mégnin and Trouessart, 1884). This type of distribution is in surprising contrast to other six genera of Kramerellidae, each of which is associated with a particular bird order (Gaud and Atyeo, 1996).

**Table 4:** Host associations of *Pseudogabucinia* species (PW – present work, * – type host).

Mite	Host	Host family	Host order	Reference
*Pseudogabucinia ciconiae* (Canestrini et Buckley et al., 2012)	*Ciconia alba*	Ciconiidae	Ciconiiformes	Canestrini and Berlese, 1881; Cerny, 1961
*P. intermedia* (Megnin et Thompson et al., 1997)	*Falco biarmicus*	Falconidae	Falconiformes	Gaud, 1983a
	Falco eleonorae*	Falconidae	Falconiformes	Mégnin and Trouessart, 1884; Gaud, 1983a
«	*Falco peregrinus*	Falconidae	Falconiformes	Gaud, 1983a
«	*F. subbuteo*	Falconidae	Falconiformes	Gaud, 1983a
«	*Buteo*	Accipitridae	Accipitriformes	Gaud, 1983a, 1988
«	*Circus aeruginosus*	Accipitridae	Accipitriformes	Dubinin, 1956
«	*C. cyaneus* (=*C. pallescens*)	Accipitridae	Accipitriformes	Dubinin, 1956
«	*C. pygargus* (=*C. cineraceus*)	Accipitridae	Accipitriformes	Dubinin, 1956
«	*Lophaetus occipitalis*	Accipitridae	Accipitriformes	Gaud, 1988
*P. microdisca* (Gaud et Mironov, 2016)	Ardeotis arabs stibieri*	Otididae	Otidiformes	Gaud and Mouchet 1961
«	*Lissotis melanogaster*	Otididae	Otidiformes	Gaud and Mouchet 1961
*P. moucheti*Gaud, 1968	*Balearica pavonica*	Gruidae	Gruiformes	Gaud, 1968
*P. nisaeti* sp. n.	*Nisaetus philippensis*	Accipitridae	Accipitriformes	PW
*P. reticulata* Atyeo et Windingstad, 1979	*Grus canadensis tabida*	Gruidae	Gruiformes	Atyeo and Windingstad, 1979

Among previously known *Pseudogabucinia* species, *Pseudogabucinia intermedia* (Mégnin et Trouessart, 1884) has been recorded from raptor birds of two orders: from falcons *Falco* (Falconiformes: Falconidae), harriers *Circus* (Accipitriformes: Accipitridae) and buzzards *Buteo* (Gaud, 1988). Association of one species on hosts from different orders is quite rare among feather mites; therefore, it cannot be excluded that *P. intermedia* from these hosts (Table 4) could represent separate species. In the differential diagnosis below, the new species is compared with the specimens of *P. intermedia* from falcons.

Type material. Male holotype (ZISP 7307), 4 male and 1 female paratypes from *Nisaetus pinskeri* (Gould, 1863). (Accipitridae), THE PHILIPPINES, Salaysay, Davao City, caught in 2005, mite collector O.O. Tolstenkov. Voucher specimen: female paratype ZISP 7312.

Depository. Holotype, 3 male and 1 female paratypes – ZISP, 1 male paratype UMICHZ.

**Fig. 7:**
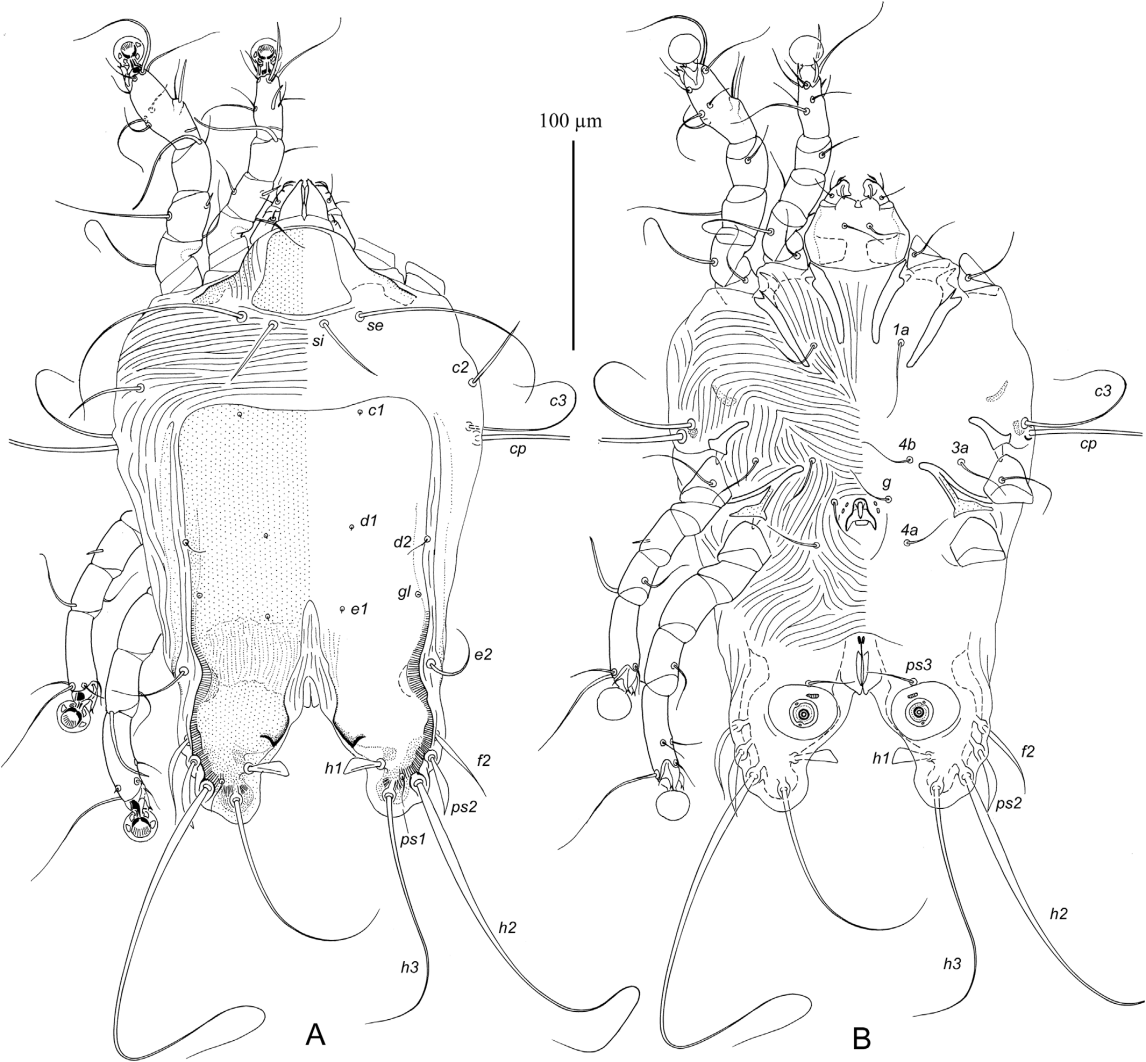
*Pseudogabucinia nisaeti* sp. n. male. A – dorsal view, B – ventral view.

MALE (Fig. 7, 9A-E). (Holotype, range for three paratypes in parentheses). Gnathosoma: length including palps 43 (42–45), greatest width at base 50 (48–52). Idiosoma length from anterior end to lobar apices (bases of setae *h3*) 270 (265–280), greatest width at level of humeral setae 175 (170–180); length of hysterosoma 195 (190–195). Prodorsal shield: occupying anterior part of prodorsum, roughly trapezoidal in shape, with slightly convex posterior margin and posterior angles slightly extending laterally, not extending to bases of scapular setae, length along midline 45 (45–48), greatest width 47 (45–50) (Fig. 7). Setae *se* separated by 57 (55–58). Setae *si* spiculiform, 35 (35–47) long, separated by 23 (22–25), approximately equidistant from midline and corresponding setae *se*. Scapular and humeral shields absent. Setae *c2* spiculiform, 30 (27–32) long, situated in striated tegument. Subhumeral setae long filiform, nearly half the length of macrosetae *cp.* Hysteronotal shield: greatest length from anterior margins to bases of setae *h3* 185 (180–190), width at anterior margin 125 (115–125), anterior margin slightly concave, lateral margins almost straight, surface with fine longitudinal striae between levels of setae *e1* and *e2*. Supranal concavity narrowed anteriorly and extending to level of setae *e1*. Hysteronotal gland opening *gl* situated approximately equidistant from levels of setae *d2* and *e2*. Lateral bands poorly demarcated. Seta *d2* minute filiform, about 5 long, setae e2 filiform 32 (27–33). Opisthosomal lobes roughly triangular, with rounded posterior ends, approximately as long as wide at base; apical and inner margins of lobes membranous. Terminal cleft wide triangular, with blunt anterior very end, 52 (52–55) long, 52 (50–55) in width at level of setae *h3*. Setae *f2* narrowly lanceolate with short filiform apex 40 (32–40); setae *ps2* blade-shaped 27 (25–27); setae *h1* narrowly triangular, 15 (15–18) long thin, setae *ps1* filiform, about 10 long, situated posterior to level of setae *h1*. Distances between dorsal setae: *c2:d2* 77 (70–80), *d2:e2* 57 (55–60), *e2:h3* 63 (57–63), *d1:d2* 37 (35–40), *e1:e2* 25 (22–28), *f2:f2* 112 (110–120), *ps1:ps1* 85 (82–88), *h3:h3* 72 (70–75), *h2:h2* 105 (100–105).

Epimerites I free, slightly converging. Epimerites IIa present, barely distinct. Genital apparatus 15 (14–15) in length, 13 (13–17) in width, its base situated at midlevels of trochanters IV (Fig. 7B). Setae *3a* and *4b* situated at the same level. Setae *g* at level of apex of genital arch. Genital papillae situated lateral to anterior half of genital arch. Distances between ventral setae: *4b:g* 17 (16–18), *g:4a* 23 (20–23); *4a:ps3* 62 (60–64), *ps3:h3* 47 (47–50). Anal suckers 13 (12–14) in diameter, corolla with two rounded denticles. Small adanal sclerites presents between setae *ps3* and anal suckers.

Solenidion *σ1* of genu I approximately half the length of this segment. Setae *mG* of genu II much longer than of genu I. Setae *cG* of genua I and III filiform, slightly longer than corresponding segments. Solenidion *φ* of tibia IV slightly shorter than tarsus IV. Setae *d* and *e* of tarsi IV minute spine-like. Legs IV with ambulacral disc slightly extending beyond level of lobar apices. Length of tarsi: I 33 (32–34), II 42 (40–43), III 40 (37–40), IV 43 (40–43). Length of solenidia: *σ1*I 5 (5–6), *σ*II4 (4–6), *σ*III 5 (5–6), *ω1*I 12 (12–14), *ω1*II 20 (18–20).

**Fig. 8:**
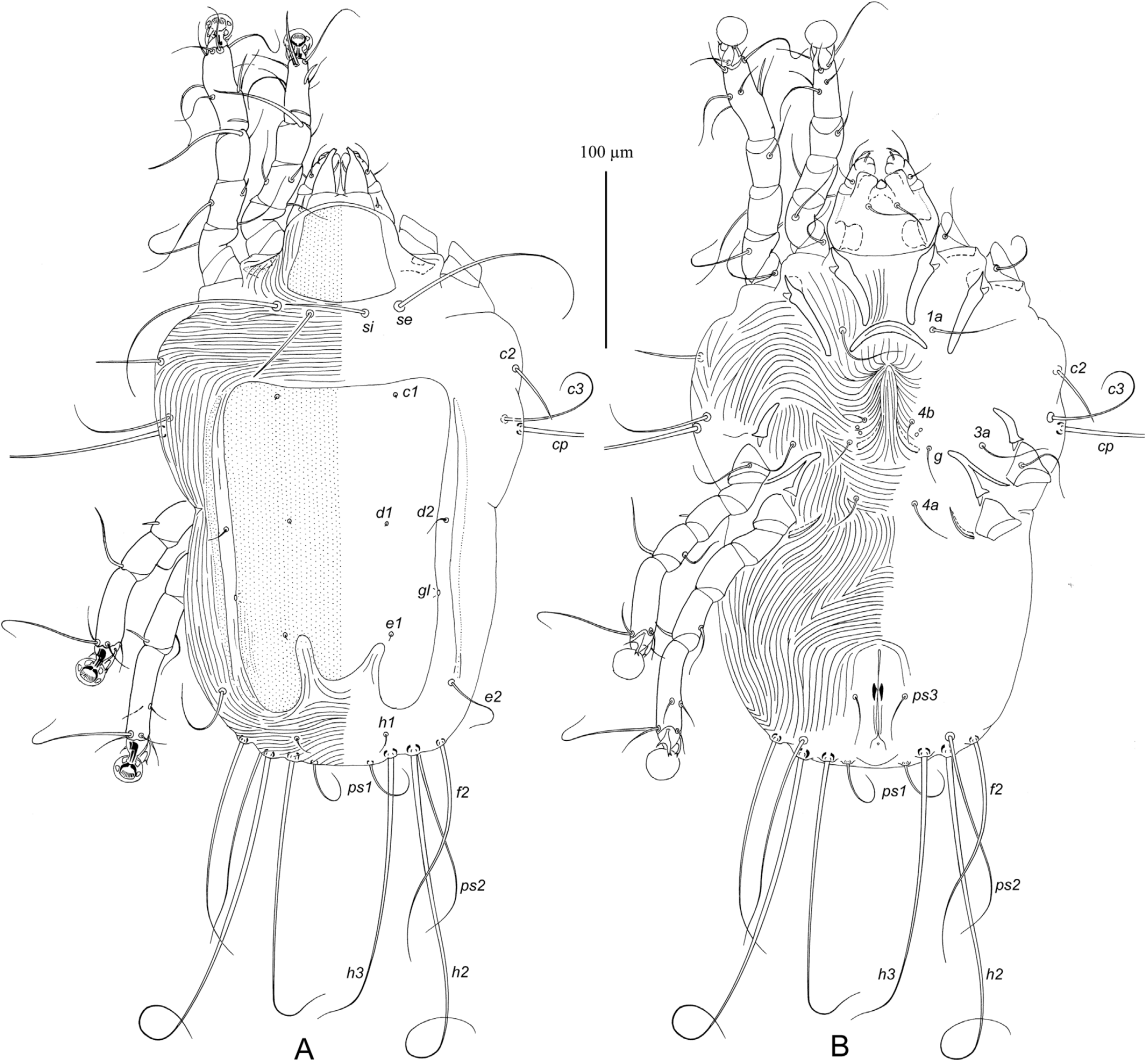
*Pseudogabucinia nisaeti* sp. n. female. A – dorsal view, B – ventral view.

**Fig. 9:**
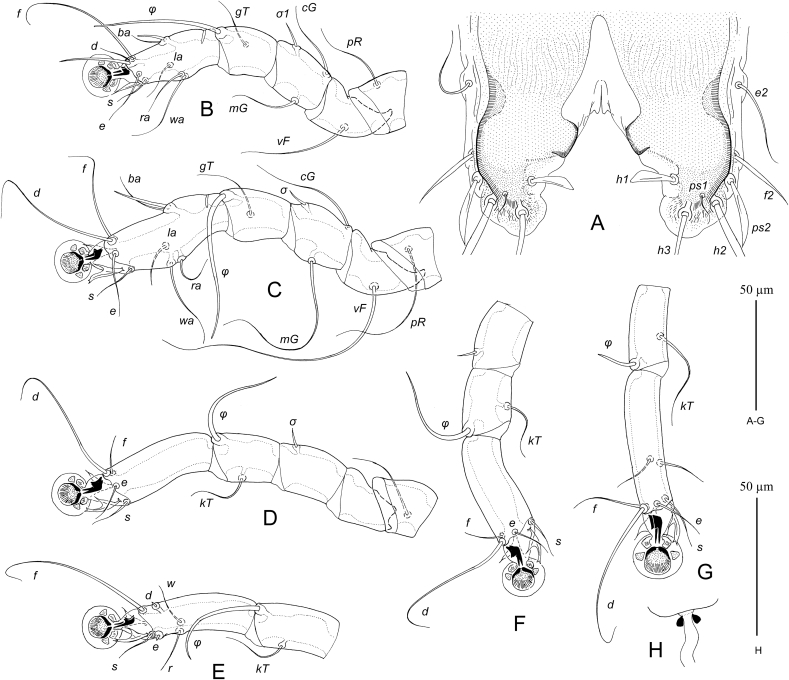
*Pseudogabucinia nisaeti* sp. n. details. A – opisthosoma of male, ventral view, B–D – genu, tibia and tarsus I–III of male, respectively, dorsal view, E – tibia and tarsus IV of male, F, G – tibia and tarsus III and IV of female, respectively, G – tibia and tarsus IV of female, H – spermatheca and spermaducts.

FEMALE (Fig. 8). Gnathosoma, length × width, 55 × 63. Idiosoma, length × width, 310 × 200, length of hysterosoma 230. Prodorsal shield: shaped as in male, 55 × 58. Setae *se* separated by 68; setae *si* spiculiform, 45 long, separated by 30, situated approximately equidistant from midline and corresponding setae *se*. Scapular and humeral shields absent. Setae *c2* thin spiculiform, 35 long, situated in anterior angles of humeral shields. Subhumeral setae *c3* long filiform 37 long, about half the length of setae *cp*. Hysteronotal shield: length 180, width 125, anterior margin nearly straight, not extending to level of setae *c2*, surface without ornamentation, posterior margin with pair of narrow incision almost extending to level of setae *e1* and wide semi-rounded extension between them. Setae *d2* off hysteronotal shield. Lateral bands present, poorly demarcated. Spermatheca and spermaducts as in Fig. 9H, secondary spermaducts heavily sclerotized. Length of opisthosomal setae: *e2* 38, *f2* 125, *ps1* 40, *ps2* 155, *h1* 10. Distances between dorsal setae: *c2:d2* 87, *d2:e2* 83, *e1:e2* 20, *h1:h1* 50, *h2:h2* 83, *h3:h3* 55, *ps1:ps1* 32.

Epimerites I as in male. Epigynum bow-shaped, situated between tips of epimerites II, 15 long, 40 wide. Apodemes of oviporus barely sclerotized. Setae g and 3a situated approximately at same level of setae. Distances between ventral setae: *4b:g* 12, *4b:3a* 15, *g:4a* 32. Legs I–III as in male. Solenidion *φ* of tibia III slightly longer than corresponding tarsus; solenidion *φ* of tibia IV about one third the corresponding tarsus. Legs IV with ambulacral disc extending beyond posterior end of the opisthosoma.

Length of tarsi: I 35, II 50, III 45, IV 50. Length of solenidia: *σ1*I 10, *σ*II 9, *σ*III 6, *ω1*I 12, *ω1*II 20.

**Differential diagnosis**. The new species, *Pseudogabucinia nisaeti* sp. n. is close to *P. intermedia* (Mégnin et Trouessart, 1884) known from falcons by in having, in both sexes, ambulacral discs of tarsi IV extending to or slightly beyond the posterior margin of the body, and setae *c2* exceeding the distance between internal scapular setae *si*, and, in females, setae *f2* and *ps2* being equal to or exceeding the distance between their bases. *Pseudogabucinia nisaeti* sp. n. differs from that species by the following features: in both sexes, subhumeral setae *c3* are long filiform and approximately half as long and humeral setae *cp*, solenidion *ω1* of tarsus II does not extend to the apex of this segment; in males, the supranal concavity does not extend beyond the level of setae *e1*, setae *4a* are situated posterior to the base of the genital arch; in females, the genital papillae are situated distinctly anterior to the level of setae *g*. In both sexes of *P. intermedia*, subhumeral setae *c3* are about 1/3 the length of setae *cp*, solenidion ω1 of tarsus II extends to the apex of this segment; in males, the supranal concavity extend far beyond the level of setae *e1*, setae *4a* are situated posterior at the of the level of genital arch base; in females, the genital papillae are situated at the level of setae *g*.

**Etymology**. The specific epithet is derived from the generic name of the type host and is a noun in the genitive case.

### 3.3 Molecular phylogenetics

**Fig. 10:**
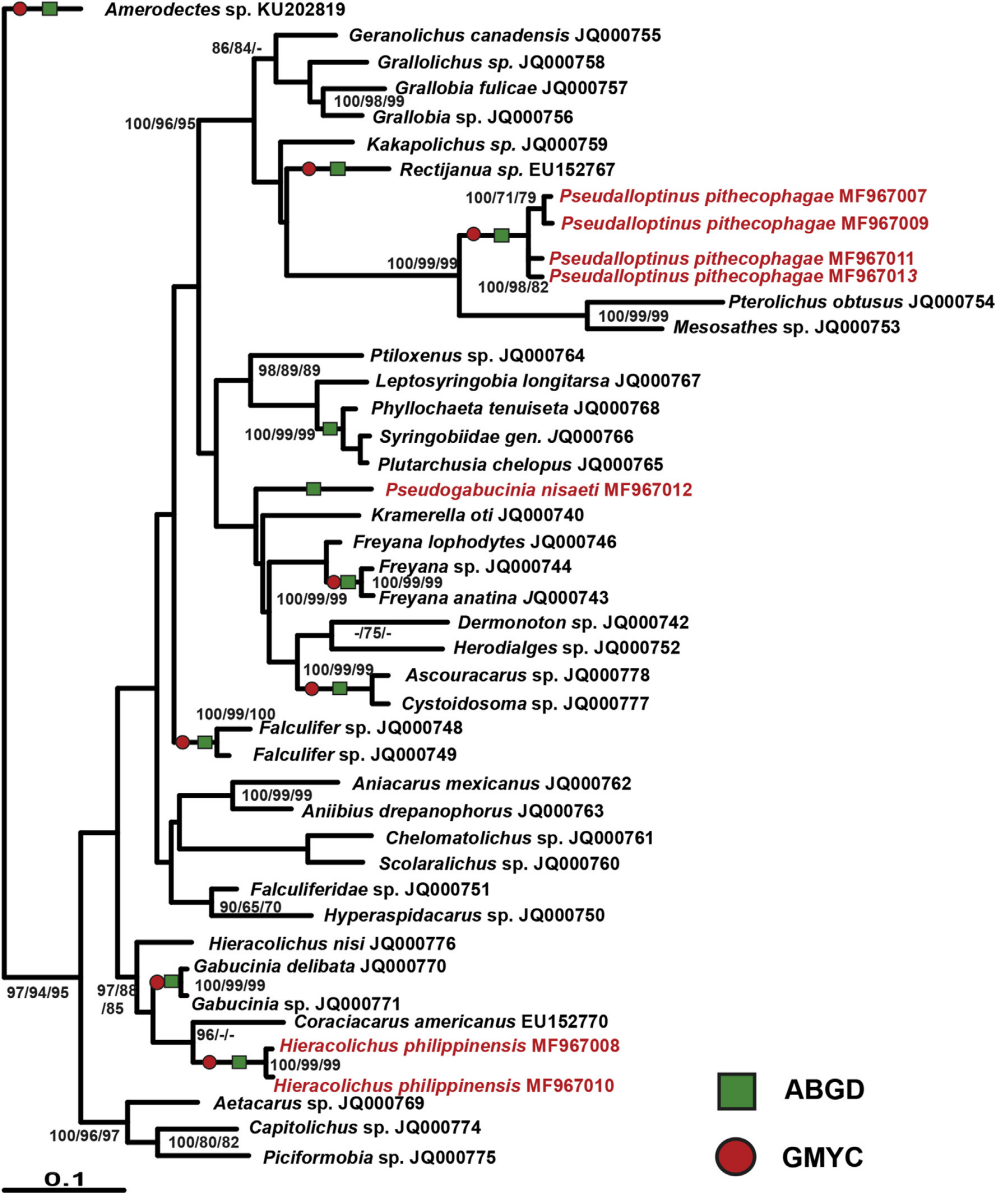
Phylogenetic tree of EF1 sequences from pterolichoid feather mites available in GenBank (black) with feather mites from Philippine raptors studied (red); tree topology was reconstructed in the RaxML program. Values of the statistical support (are given above the branches if they exceed 65%) were computed by following methods: Mr. Bayes/ML (by RaxML) and NJ (by Mega6). ABGD and GMYC marks represent significant nodes (p < 0.05). (For interpretation of the references to colour in this figure legend, the reader is referred to the Web version of this article.)

We obtained sequences for the genes COI, EF-1α, 18S, 28S from four specimens of *P. pithecophagae*, two specimens of *H. philippinensis* and one specimen of *P. nisaeti* (Table 2). Data on different molecular markers studied for feather mites of superfamily Pterolichidae in GenBank are both sparse and variable in coverage (Klimov and OConnor, 2008). Therefore, we did not provide the resulting phylogenetic tree for COI because there were very few sequences for pterolichoid feather mites available in GenBank. Phylogenetic trees for EF-1α, 18S and 28S molecular markers placed the sequences of the Philippine raptor feather mites studied among the other pterolichoid feather mites (Fig. 10, Figure S1, Figure S2). Although only the phylogenetic tree for elongation factor 1 alpha sequences showed congruent topologies between Bayesian and maximum likelihood analyses (Fig. 10). Operational taxonal unit testing analysis by both GMYC and ABGD algorithms supported delimitation of OTU hypothesized by morphological studies for feather mites *P. pithecophagae* and *H. philippinensis* from *Pithecophaga jefferyi* while for *P. nisaeti* only the ABGD delimitation was significant, which can be explained by the presence of single specimen of the latter species available for analysis.

## 4 Discussion

Most of the birds species host several groups of ectosymbionts, including obligatory feather mites and chewing lice species (Mironov, 2016; Price et al., 2003). However, our examination of captive Philippines Eagles revealed feather mites species and no chewing lice were detected. Although we sampled only three individuals of the Great Philippine Eagles, the fact that we found no chewing lice suggests that these insects are much more susceptible to the antiparasite treatment, and endemic lice will likely not survive on captive birds in the Philippine Eagle Center. Loss of chewing lice is not unusual for small populations of endangered species of birds conserved and bred in captivity (Dunn et al., 2009). The feather mites according to results of our examination are capable of surviving annual antiparasitic treatments for a long time. For example, one of the examined birds, named Thor, was captured in the wild in 1974 and at the day of examination in 2016 hosted a viable population of *H. philippinensis*. This fact, assuming this population of mites is endemic, suggests that these ectosymbionts have been able to survive 43 years in captivity.

We describe for the first time feather mites of two endangered Philippine eagles, which, if they prove to be species-specific, are endangered species too. Based on the phylogenetic position of the species described herein and known reference data on associations of their genera and families, it is possible to drawn out very preliminary hypotheses on the origin of the examined feather mite species from eagles of the Philippines. Of 16 genera of the family Gabuciniidae, eight genera, including the genus Hieracolichus, are restricted to birds of the order Accipitriformes (Gaud, 1983b; Gaud and Atyeo, 1974; Mironov et al., 2007). Most representatives of the genus Aetacarus, with exception of a few species, are associated with raptors. Although the primary origin of the family Gabuciniidae as developing on Accipitriformes is not completely proven, gabuciniids have a maximum of diversity in genera and species on Accipitriformes compared to its other host orders, like Coraciiformes, Caprimulgiformes, and Otidiformes. In any case, it is possible to state that the core of the family Gabuciniidae likely arose on the ancestors of the order Accipitriformes and extensively evolved on these birds. In this light, it is possible to suggest that *Hieracolichus philippinensis* represents the original feather mite fauna on the Great Philippine Eagle rather than a recently acquired feather mite species.

Currently the suprageneric system of the family Pterolichidae is not fully developed (Mironov, 2016). Our attempts to study the molecular phylogeny of the family showed a lack of available sequences in GenBank for many molecular markers, which make it difficult to build a reasonable concatenated tree. Nevertheless, based on the distribution of the genus Pseudalloptinus exclusively inhabiting Accipitriformes (Dubinin, 1956; Gaud, 1988), we could conclude that this genus was probably formed on the ancestors of this order and successfully evolved on these birds. In this case, like *H. philippinensis*, *Pseudalloptinus pithecophagae* also represents rather ancient and most likely the primary fauna on the Great Philippine Eagle.

Wide and mosaic distribution of the kramerellid genus Pseudogabucinia among birds orders and within Accipitriformes and Falconiformes (Table 4) strongly contrasts with other genera of the family Kramerellidae that are each restricted to a particular host order (Gaud and Atyeo, 1996). Distribution of Pseudogabucinia representatives on phylogenetically distant genera of raptors of two orders allows us to hypothesize that species associated with accipitriforms could represents some remnants of formerly rich fauna of Pseudogabucinia on these birds. On the other hand, mites of this genus could represent invading fauna transferred from other unknown host groups or rather, a transferrable mite grouping between accipitriform and even falconiform hosts.

The Great Philippine Eagles were historically placed in the subfamily Harpiinae related to other eagles but were recently moved to the family Circaetinae based on molecular studies (Lerner and Mindell, 2005; Ong et al., 2011). Although the host distribution of the genus Hieracolichus is not yet well explored, its preferential occurrence on rather basal lineages (see Lerner and Mindell, 2005) of accipitriforms, such as Aegypiinae, Circaetinae, Polyboroidinae (Gaud, 1983b; Philips, 2000), can be considered as additional evidence that *P. jefferyi* indeed belongs to the lineage of serpent eagles Circaetinae, rather than derived lineages of typical eagles as Aquilinae and Harpiinae.

## 5 Conclusions

We showed that a small captive group of endangered birds could maintain viable populations of native feather mites, demonstrating the utility of ectosymbiont examination for host individuals even after decades in captivity. We provided the first record of feather mites from endemic raptors or diurnal birds-of-prey in the Philippines, with three new feather mite species described, and revealed the native origin of the feather mites studied. Our work facilitated an understanding of biodiversity in the understudied family of feather mites Pterolichidae, although many more species should be sequenced before the relations in the family can be resolved clearly by molecular phylogeny.

## Appendix A Supplementary data

Data profile Data profile

Fig. s1 Phylogenetic tree of 18S sequences from pterolichoid feather mites available in GenBank (black) with feather mites from Philippine raptors studied (red); tree topology was reconstructed in the RaxML program. Values of the statistical support (are given above the branches if they exceed 65%) were computed by following methods: Mr. Bayes/ML (by RaxML) and NJ (by Mega6). ABGD and GMYC marks represent significant nodes (p < 0.05). Fig. s1

Fig. s2 Phylogenetic tree of 28S sequences from pterolichoid feather mites available in GenBank (black) with feather mites from Philippine raptors studied (red); tree topology was reconstructed in the RaxML program. Values of the statistical support (are given above the branches if they exceed 65%) were computed by following methods: Mr. Bayes/ML (by RaxML) and NJ (by Mega6). ABGD and GMYC marks represent significant nodes (p<0.05). Fig. s2
