# Supplementary figures and images for: Captive individuals of endangered Philippine raptors maintain native feather mites (Acariformes: Pterolichoidea) species
Source: Int J Parasitol Parasites Wildl. 2018 Mar 17;7(2):116–33. doi: 10.1016/j.ijppaw.2018.03.002 (PMC6031967; doi:10.1016/j.ijppaw.2018.03.002)

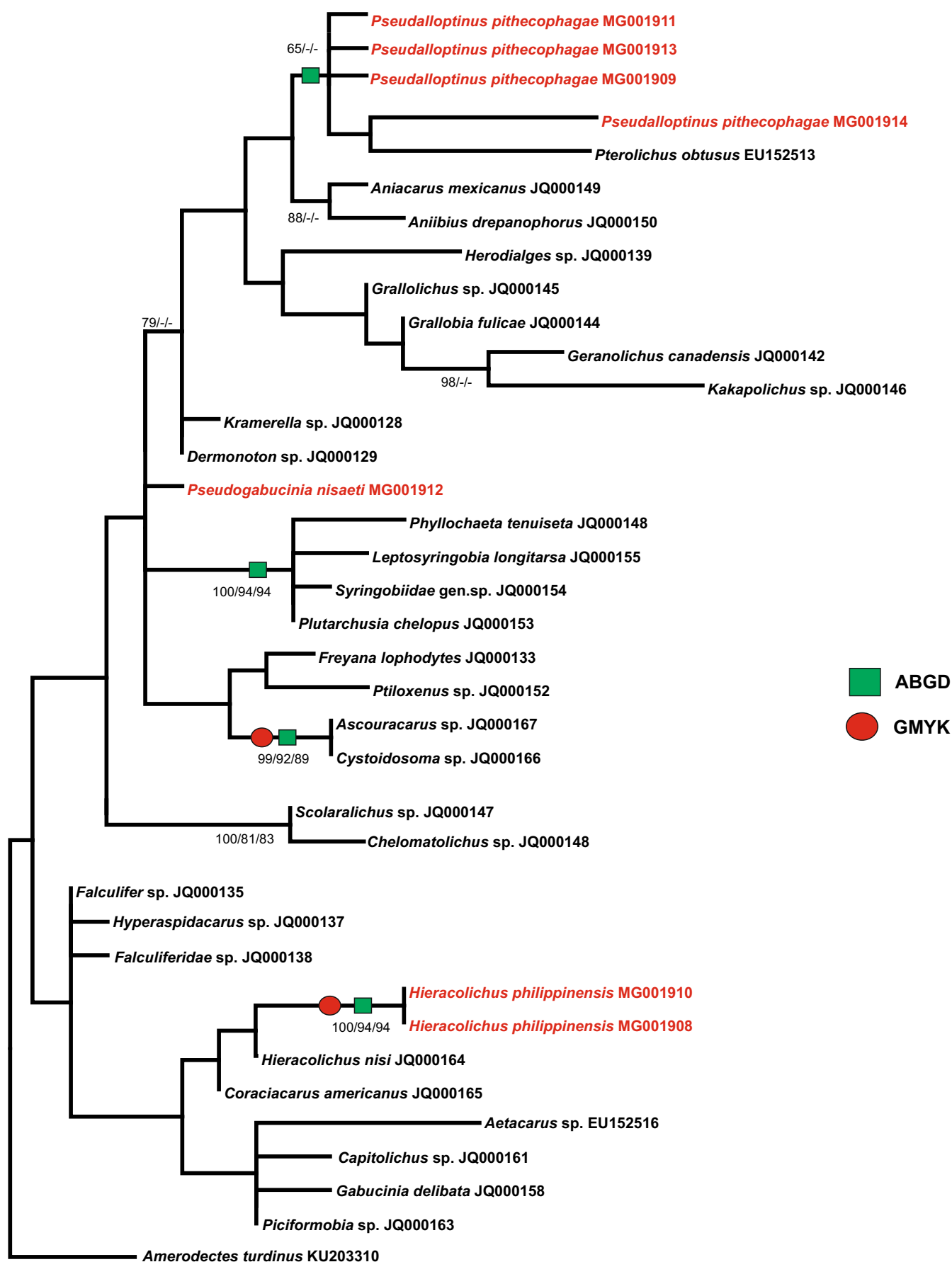

0,005

Supplement: Fig. s1 — Phylogenetic tree of 18S sequences from pterolichoid feather mites available in GenBank (black) with feather mites from Philippine raptors studied (red); tree topology was reconstructed in the RaxML program. Values of the statistical support (are given above the branches if they exceed 65%) were computed by following methods: Mr. Bayes/ML (by RaxML) and NJ (by Mega6). ABGD and GMYC marks represent significant nodes (p < 0.05). [file mmc2.pdf]

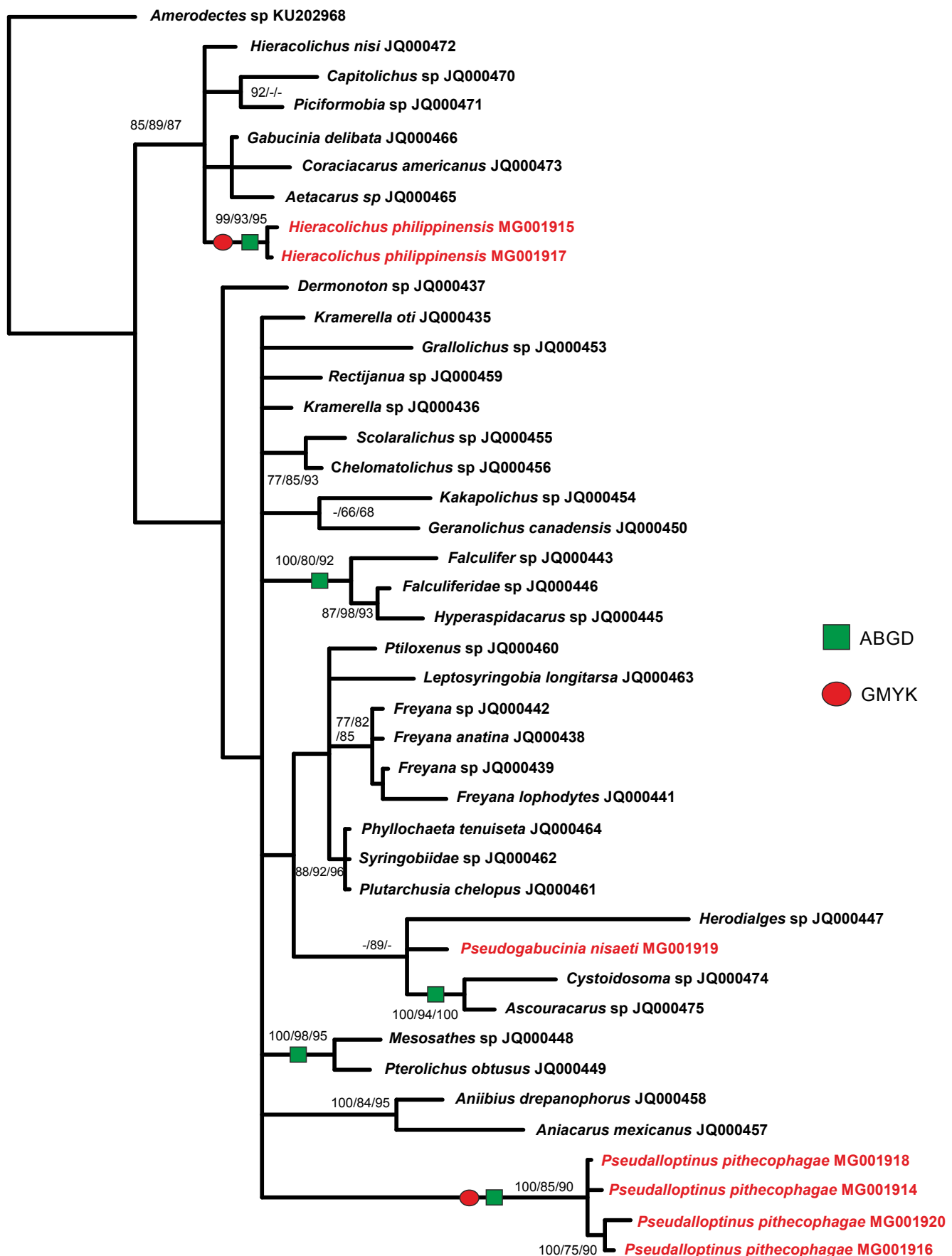

Supplement: Fig. s2 — Phylogenetic tree of 28S sequences from pterolichoid feather mites available in GenBank (black) with feather mites from Philippine raptors studied (red); tree topology was reconstructed in the RaxML program. Values of the statistical support (are given above the branches if they exceed 65%) were computed by following methods: Mr. Bayes/ML (by RaxML) and NJ (by Mega6). ABGD and GMYC marks represent significant nodes (p<0.05). [file mmc3.pdf]
